# Self-administered versus lymphedema therapist-administered complex decongestive therapy protocol in breast cancer-related lymphedema: a non-inferiority randomized controlled trial with three-month follow-up

**DOI:** 10.1007/s10549-025-07709-3

**Published:** 2025-05-06

**Authors:** Sukriye Cansu Gultekin, Didem Karadibak, Ahmet Burak Cakir, Zeynep Gulsum Guc, Tugba Yavuzsen

**Affiliations:** 1 Faculty of Physiotherapy and Rehabilitation, Graduate School of Health Sciences, Dokuz Eylul University, Izmir, Turkey; 2 Faculty of Physiotherapy and Rehabilitation, Department of Physiotherapy and Rehabilitation, Division of Cardiopulmonary Physiotherapy-Rehabilitation, Dokuz Eylul University, Izmir, Turkey; 3 Department of Medical Oncology, Izmir Katip Celebi University Ataturk Training and Research Hospital, Izmir, Turkey; 4 Faculty of Medicine, Department of Internal Medicine, Division of Oncology, Dokuz Eylul University, Izmir, Turkey

**Keywords:** Breast cancer, Upper extremity lymphedema, Complex decongestive therapy, Self-care, Health parameters

## Abstract

**Purpose:**

The aim of this study was to demonstrate that a self-administered complex decongestive therapy (CDT) protocol is not inferior to certified lymphedema therapist (CLT)-administered CDT in the management of lymphedema and health-related outcomes in patients with breast cancer-related lymphedema (BCRL).

**Methods:**

Fifty patients with BCRL were randomly assigned to two experimental groups: (1) a CLT-administered CDT group (*n* = 25) or a self-administered CDT group (*n* = 25). CDT was a multimodal approach in two experimental conditions consisting of patient education, manual lymph drainage, multi-layer bandaging, therapeutic exercises and skin/nail care. Lymphedema severity was assessed using circumference measurement, and BCRL-related symptoms were assessed using a numerical rating scale. The following measurement methods were used to assess health-related outcomes: universal goniometer for range of motion (ROMs), hand grip dynamometer for peripheral muscle strength, disabilities of the arm, shoulder and hand (DASH) questionnaire for disability level, International Physical Activity Questionnaire-Short Form (IPAQ-SF) for physical activity level, the checklist for individual strength (CIS) for fatigue and upper limb lymphedema quality of life questionnaire (ULL-27) for quality of life.

**Results:**

Following CDT, there was a significant decrease in lymphedema severity and lymphedema-related symptoms in both groups (*p* < 0.001). There was no significant difference between the groups regarding the mean difference in health-related outcomes following CDT (post-treatment-baseline) (*p* < 0.05). Lymphedema severity and symptoms remained stable during the 3-month follow-up periods in the CLT-administered CDT group (*p* > 0.05). There was a decrease in the severity of lymphedema, stiffness, heaviness and fatigue in the self-administered CDT group at 3-month follow-up (*p* < 0.05), while pain and tingling remained unchanged (*p* > 0.05).

**Conclusion:**

The present findings demonstrated self-administered CDT protocol is not inferior to CLT-administered CDT in the management of lymphedema and reduction of lymphedema-related disabilities.

**Graphical Abstract:**

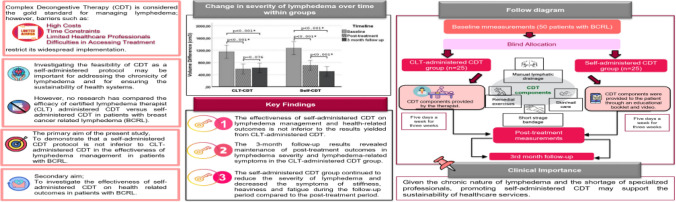

**Supplementary Information:**

The online version contains supplementary material available at 10.1007/s10549-025-07709-3.

## Introduction

Breast cancer (BC) is the most common type of cancer in women worldwide and its burden has been increasing in recent years [1]. Improvements in screening methods and adjuvant therapies have reduced breast cancer mortality rates and increased BC survival rates to over 90% [1]. Higher survival rates have shifted the focus on the short- and long-term to better management of side effects related to BC treatments [2]. Breast cancer-related lymphedema (BCRL), a lifelong risk in BC survivors, is an incurable but manageable sequelae of BC [3]. BCRL is characterized by the accumulation of protein-rich fluid in the interstitial space of the affected extremity, trunk and breast tissues, accompanied by fibro-adipose tissue changes [3]. The symptoms of BCRL usually include arm tightness, heaviness/fullness, pain, fatigue and extremity dysfunction, which may severely impact the daily functioning of patients [4]. Currently, no accepted medical and surgical approach exists for the treatment of BCRL. Thus, this condition remains a major concern for BC patients and healthcare professionals [3].

Complex decongestive therapy (CDT) is considered the "gold standard" for lymphedema management [5]. CDT consists of 2 phases: Phase I focuses on reducing lymphedema through intensive treatments including patient education, multi-layer bandaging, manual lymph drainage (MLD), therapeutic exercises and skin/nail care. Phase II shifts to maintaining the improvements obtained in Phase I with compression garments, self MLD application, exercises and skin/nail care to provide long-term symptom control [5]. Studies have reported CDT to reduce lymphedematous extremity volume by 30% to 50% in BCRL patients and to be beneficial in management of symptoms [6–8]. On the other hand, the high cost of CDT, its time-intensive application, the shortage of trained healthcare professionals and limited accessibility for patients in remote areas present significant barriers to participation in the CDT programme [8–10]. Furthermore, BCRL is a chronic condition, and extremity volume may fluctuate due to various factors such as temperature, atmospheric pressure changes, insect bites and daily activities. Consequently, patients may require multiple referrals to the CDT programme throughout their lifetime [5, 11].

Lymphedema is recognized by the World Health Organization (WHO) as a public health problem in third world countries and highlighted the difficulties in the management of lymphedema and stated that self-care protocols should be adopted by patients [12]. Additionally, the National Cancer Institute (NCI) has emphasized the critical importance of addressing chronicity in cancer rehabilitation, promoting self-care and ensuring the sustainability of health systems in supportive care [13]. Therefore, identifying beneficial practices transferable across cultural and economic boundaries might emerge as an important approach to reduce healthcare costs and improving the long-term health outcomes of patients with lymphedema [12, 14]. Previous studies have investigated the efficacy of self-care protocols separately, including patient education, self-exercise programmes, self-administration of MLD and the use of compression garments [14, 18–20]. However, none of these studies compared the efficacy of self-administration of Phase I of CDT adopting a multimodal approach (MLD, skin/nail care, multi-layer bandaging and exercises) with certified lymphedema therapist (CLT)-administered CDT. Additionally, even though previous self care studies reported improvements in disease-related disabilities levels changes to a degree, their findings were inferior to the beneficial effects obtained through a CLT-administered CDT programme [10, 15, 16]. Therefore, the primary aim of this study was to demonstrate that a self-administered CDT protocol (including all aspects of CDT) is not inferior to a CLT-administered CDT in lymphedema management (lymphedema severity and lymphedema-related symptoms). Secondary aims were to investigate the effectiveness of self-CDT on health-related outcomes (upper limb functional outcomes, physical activity level, fatigue, patient benefit index, health-related quality of life (HRQoL)) in patients with BCRL.

## Materials and methods

### Study design

This study used a parallel, prospective, single-blind randomized controlled study design. Patients referred from the Department of Medical Oncology of Dokuz Eylul University Hospital and the Department of Medical Oncology of Izmir Katip Celebi University Atatürk Training and Research Hospital to the Department of Rehabilitation of Dokuz Eylul University Faculty of Physical Therapy were enrolled. The present study was carried out between January 2024 and November 2024. Ethical approval of the study was obtained from Dokuz Eylul University Ethics Committee for Non- Invasive Studies (decision number: 2023/34-09, date: 25.10.2023). All procedures were performed in accordance with the Declaration of Helsinki. The study procedure was recorded, and a registration number was assigned (clinicaltrials.gov, study number: NCT06652295 registration date: 19.10.2024). The informed consents were obtained from all participants.

### Participants

Based on the inclusion and exclusion criteria, 50 women with BCRL volunteered to be included in the study. The inclusion criteria were as follows: (1) being 18 years of age or older; (2) having stage 2–3 lymphedema according to the International Society of Lymphology (ISL) classification; (3) having a diagnosis of unilateral BCRL (Doppler, lymphoscintigraphy, and computed tomography were preferred to confirm the diagnosis of lymphedema based on a comprehensive medical assessment and condition of the patient); (4) no evidence/suspicion of cancer recurrence after completion of adjuvant local and systemic therapies for at least 12 months; (5) not having received a CDT programme in the last 12 months; (6) volunteering to participate in the study; and (7) being able to read and understand Turkish. Exclusion criteria were as follows: (1) history of congenital lymphedema or bilateral upper extremity lymphedema or malignant lymphedema; (2) presence of neurological or mental illness or axillary web syndrome, major organ failure and/or iatrogenic disease (such as use of steroids, nonsteroidal anti-inflammatory drugs and calcium channel blockers) that may adversely affect the severity of lymphedema; (3) presence of conditions contraindicated for CDT (active infection, deep vein thrombosis/thrombophlebitis, cardiac oedema, pulmonary disease, peripheral arterial disease, any skin disease such as scleroderma, allergic reactions to treatment); (4) not participating in three consecutive sessions or attending less than 80% of the total sessions; and (6) being previously enrolled in a self-physiotherapy programme (self MLD, self-multiple-layer short stretch bandaging, self exercises or a combination of these).

### Sample size

The sample size was calculated by a priori sample size analysis section of G*Power 3.1.9.4 (Kiel University, Düsseldorf, Germany). Using the effect size of change in lymphedema severity (0.92) from a previous study comparing the effectiveness of standard care and CDT on the severity of lymphedema between groups in patients with BCRL [17], a type I error rate of 5% and a minimum power of 85%, required minimum sample size was determined to be 46 BCRL patients. This number was increased by 10% to account for potential drop-outs during the study period, yielding a total of 50 patients with BCRL (25 in each group, with an allocation ratio of 1:1).

### Procedures

A structured form was used to record demographic information (age, gender, height, weight, BMI, married status, working status, affected extremity dominancy, comorbidities, smoking status and history of CDT treatment) and disease-specific medical history (BC stage and histological subtype, BC-free survival time, lymphedema stage, duration of lymphedema disease, history of BC surgery and adjuvant local and systemic therapies) for each participant. All baseline assessments of patients were performed by a blinded researcher prior to randomization. The block randomization method was employed using the Randomizer software program [18], and participants were assigned to one of two experimental groups (CLT-administered CDT or self-administered CDT). All clinical parameters were assessed at baseline and post-treatment (following 15 sessions of CDT). At the 3-month follow-up, the primary outcome measures (arm circumference (lymphedema severity) and lymphedema-related symptoms) were re-evaluated. All assessments (baseline, post-treatment and 3-month follow-up assessments) were performed by the same blinded researcher. Following the 3-week CDT programme, participants in both groups were advised to use lymphedema-specific arm sleeves (arm sleeve pressure used compression class (CCL 2), moderate compression (23–32 mmHg). Patient education was also provided about the use of the arm sleeves (wearing, washing and cautions).

### Interventions

Following randomization, participants were assigned to one of two experimental groups: (1) CLT-administered CDT or (2) a self-administered CDT programme. The treatment protocols for both groups were structured and carried out by a lymphedema therapist who was certified from Academy of Lymphatic Studies (ACOLS). All participants in both experimental groups received an approximately one-hour session of patient education from a CLT prior to beginning their treatment programme and were thoroughly informed about all components of CDT. Patient education focused on the following points: (1) the structure of the lymphatic system; (2) precautions for daily activities (avoidance of repetitive work, overload, etc.); (3) measures to prevent factors that adversely affect lymphedema (sun protection, prevention of injuries/mosquito bites, weight gain); (4) skin and nail care (skin washing with a low pH cleaning gel, use of low pH lotions, prevention of fungal infections, nails should be cut straight, rounded edges, etc.); (5) modifications for daily tasks (e.g. using a book holder, raising the arm during walking and pump exercises); and (6) the importance of physical activity for improving lymphatic flow and venous return. All participants were encouraged to engage in brisk walking to promote physical activity. The walking regime consisted of brisk walking five days a week for 30 min in the first week, 40 min in the second week and 45 min in the third week. The compliance of participants with the walking recommendation was followed up using a physical activity diary.

#### Clt-administered CDT group

The group received a therapist-administered CDT programme five days a week (weekdays) for three weeks. All components of the CDT were performed in the following order: MLD, application of multi-layer bandaging and therapeutic exercise programme. All applications were completed in approximately one hour (a total of 15 hour of CDT treatment was given). After 15 sessions of CDT, patients then progressed to Phase II and were instructed on the usual standard care steps (simple MLD application, use of an arm sleeve). Additionally, patients were instructed to continue their therapeutic exercise programme.

#### Self-administered CDT group

The self-administered CDT programme consisted of self-administered MLD, self-administered multi-layer bandaging application, skin/nail care and therapeutic exercises. This group was given practical information about all the applications by the lymphedema therapist for approximately two-hour in one session prior to beginning the self-administered CDT programme and the patients were provided with practice in the application. Each component of the self-administered CDT programme was also presented to the participants with a patient booklet (Appendix 1) and a video prepared by the researchers. The self-administered CDT group were instructed to perform the programme five days a week (on weekdays) for three weeks (for a total of 15 hour). Patients were followed up once a week, on the same day each week, using a patient follow-up diary to monitor manual lymphatic drainage (MLD), multi-layer bandaging, therapeutic exercises, skin/nail care and walking. Subsequent to 15 sessions, self-administered CDT was encouraged to practice it again if necessary, and patient education was provided to patients using arm sleeves.

#### Therapeutic exercises

The therapeutic exercises were designed to facilitate lymph flow, enhance muscle pump activity and improve overall physical function [5]. Therapeutic exercises were performed from distal to proximal (based on the principle of using muscle activation (muscle pump) to move lymph fluid forwards and to the venous angle). The exercises were performed in combination with breathing exercises (to promote an increase in venous return) [19]. All patients included were instructed to perform the same exercise routine. These exercises were performed once a day as 2 set of 5–7 repetitions after bandage application. The CLT-administered CDT group performed the exercises under the supervision of a therapist, while the self-administered CDT group performed the exercises at home. The therapeutic exercise routine applied in the present study is shown in Appendix 2***.***

## Primary outcome measurements

### Lymphedema severity

Circumference was measured to assess the severity of upper extremity lymphedema. All participants were assessed in the standard supine position with the arms in 30° abduction using a standard 2.54 cm (1 in) retractable fibreglass tape. The circumference of both extremities was measured every 5 cm from the third nail to proximally. Measurements of both arms were recorded in centimeters (cm). Volume differences were calculated using the frustum formula [20]. The severity of lymphedema was classified according to the criteria defined by the International Society of Lymphology (ISL): mild lymphedema, < %20; moderate lymphedema, 21 to 40%; and severe lymphedema, > 41% [5].

### BCRL-related symptoms assessment

A numerical rating scale was used for symptom assessment in BCRL patients. This scale was used to assess disease-specific symptoms (stiffness, heaviness, pain, tingling, fatigue) frequently reported in previous studies [21, 22]. The scale is scored between 0 and 10 (0 indicates absence of symptoms; 10 indicates unbearable severity of symptoms).

## Secondary outcome measurements

### Range of motion

The range of motion (ROMs) of the patients were assessed using a universal goniometer (Standard BASELINE 12-inch plastic goniometer, (Model 12–1000), New York). ROMs of all upper extremity joints including shoulder (flexion, extension, abduction, adduction, internal/external rotation), elbow (flexion–extension) and wrist (flexion–extension) were measured. Shoulder and elbow ROMs were measured in the supine position and wrist ROMs were measured in the sitting position. Goniometer measurements were performed using a standardized procedure and the measurements were recorded in degrees [23].

### Peripheral muscle strength

Hand grip strength was assessed with a Jamar hydraulic hand dynamometer (Model 5030 J1, Sammons Preston Rolyan, Bolingbrook, IL, USA), as an indicator of peripheral muscle strength. Measurements were performed using the standard position recommended by the American Society of Hand Therapists [24]. Three consecutive measurements were performed with a 1-min rest period between measurements, and highest outcome in kilograms was used for analysis.

### Upper extremity disability level

The level of upper extremity disability and the difficulty in performing daily activities were assessed using the disabilities of the arm, shoulder and hand (DASH) questionnaire [25]. The questionnaire consists of 30 questions divided into 2 subcategories: disability/symptom scores and work module score. Each item is assessed on a 5-point Likert scale (1: no difficulty and 5: impossible to complete). The total score ranges from 0 to 100, with a high score indicating more upper extremity related disability. The Turkish version of the DASH questionnaire was shown to have adequate psychometric properties [25].

### Physical activity level

Physical activity level was measured using the International Physical Activity Questionnaire-Short Form (IPAQ-SF). The IPAQ-SF questionnaire inquires the level of physical activity in the prior seven days. The questionnaire consists of seven questions about the time spent in sitting, walking, moderate-intensity activities and vigorous activities. The score is calculated by counting the minutes, days and metabolic equivalent (MET) values of the corresponding activities. Participants were also categorized into physical activity subgroups based on their IPAQ-SF scores. Physical inactivity was defined as < 600 MET-min/week, low physical activity as 600–3000 MET-min/week and adequate physical activity as > 3000 MET-min/week. [26]. The Turkish version of the IPAQ-SF has been shown to be a valid and reliable tool for assessing physical activity levels [26].

### Fatigue

The checklist for individual strength (CIS) was used to assess the level of fatigue. The questionnaire is composed of 20 items and four dimensions: subjective feeling of fatigue (eight items), motivation (four items), physical activity (three items) and concentration (five items). The answer to each question in the questionnaire ranges from 1 to 7. The total score was obtained by summing the item scores and ranged between 20 and 140 [27]. A higher score on the CIS indicates greater levels of fatigue [27]. The CIS has adequate psychometric proprieties to assess fatigue in cancer patients. The Turkish version of the CIS was found as a valid and reliable tool for assessing fatigue [27].

### Treatment benefits and goals

The Patient Benefit Index-Lymphedema (PBI-L) is a 23-item questionnaire designed to assess the effectiveness of treatment in patients with lymphedema. It consists of two subsections: Patient Needs Questionnaire (PNQ) and Patient Benefit Questionnaire (PBQ). The PNQ assesses patients' pre-treatment expectations, while the PBQ measures the extent to which these expectations are satisfied after treatment. Patients rate the importance of their treatment needs on a scale from 0 (not at all) to 4 (very much) in the PNQ. The PBQ uses the same 23 items using the same rating scale to assess whether and to what extent their expectations have been fulfilled. The total score is calculated based on the difference between the pre- and post-treatment ratings, with a possible range of 0 to 4. The questionnaire includes two subscales: first, focussing on activities of daily living and psychological well-being, which contains 14 items; second, assessing physical well-being and abilities, which contains 9 items. Turkish validity and reliability have been demonstrated in lymphedema patients [28].

### Health-related quality of life

Upper Limb Lymphedema Quality of Life Questionnaire (ULL-27) was used to assess health-related quality of life (HRQoL) of BCRL patients. The ULL-27 consists of 27 total items and covers physical, psychological and social dimensions. Each item is scored on a five-point Likert scale ranging from 1 (strongly disagree) to 5 (strongly agree). Items 1–15 evaluate the physical dimension (scores ranging from 15 to 75), items 16–22 evaluate the social dimension (scores ranging from 7 to 35) and items 23–27 evaluate the psychological dimension (scores ranging from 5 to 25) [29]. Overall and subscores are calculated by summing the individual item scores and are adjusted to a score between 0 and 100 for all global and subscale scores, with higher scores indicating a worse quality of life. ULL-27 has validity and reliability in Turkish language [29].

### Statistical analysis

The statistical analyses were performed using IBM SPSS Statistics for Windows (version 20.0, Armonk, NY: IBM Corp.). Shapiro–Wilk test, histograms, detrended-Q-Q plot graphs, kurtosis and skewness values were screened to examine the normal distribution. Continuous data were expressed as mean, standard deviation or 95% confidence interval for normally distributed variables, and median and interquartile range between 25 and 75th quartiles (IQR 25/75) for non-normally distributed data. A paired samples t-test was used for normally distributed data, while the Wilcoxon signed-rank test was applied for non-normally distributed data to assess within-group changes from baseline to post-treatment. Between-group comparisons of baseline-to-post-treatment changes were conducted using the independent samples t-test (Student's t-test) for normally distributed data or the Mann–Whitney U-test for non-normally distributed data repeated-measures ANOVA was used to analyse time-dependent changes in primary outcomes across assessment time points. Chi-square test or Fisher's exact test was used for the comparison of categorical data. Statistical significance was set at *p* < 0.05.

The effect sizes (d) were estimated using means and standard deviations for normally distributed data and Z-scores for non-normally distributed data. Effect sizes were interpreted as small (*d* = 0.10–0.29), medium (*d* = 0.30–0.49) or large (*d* = 0.50–1.00) [30]. Measurement results for participants not participating in post-treatment or follow-up measurements, and intention-to-treat analyses were performed.

## Results

**Fig. 1:**
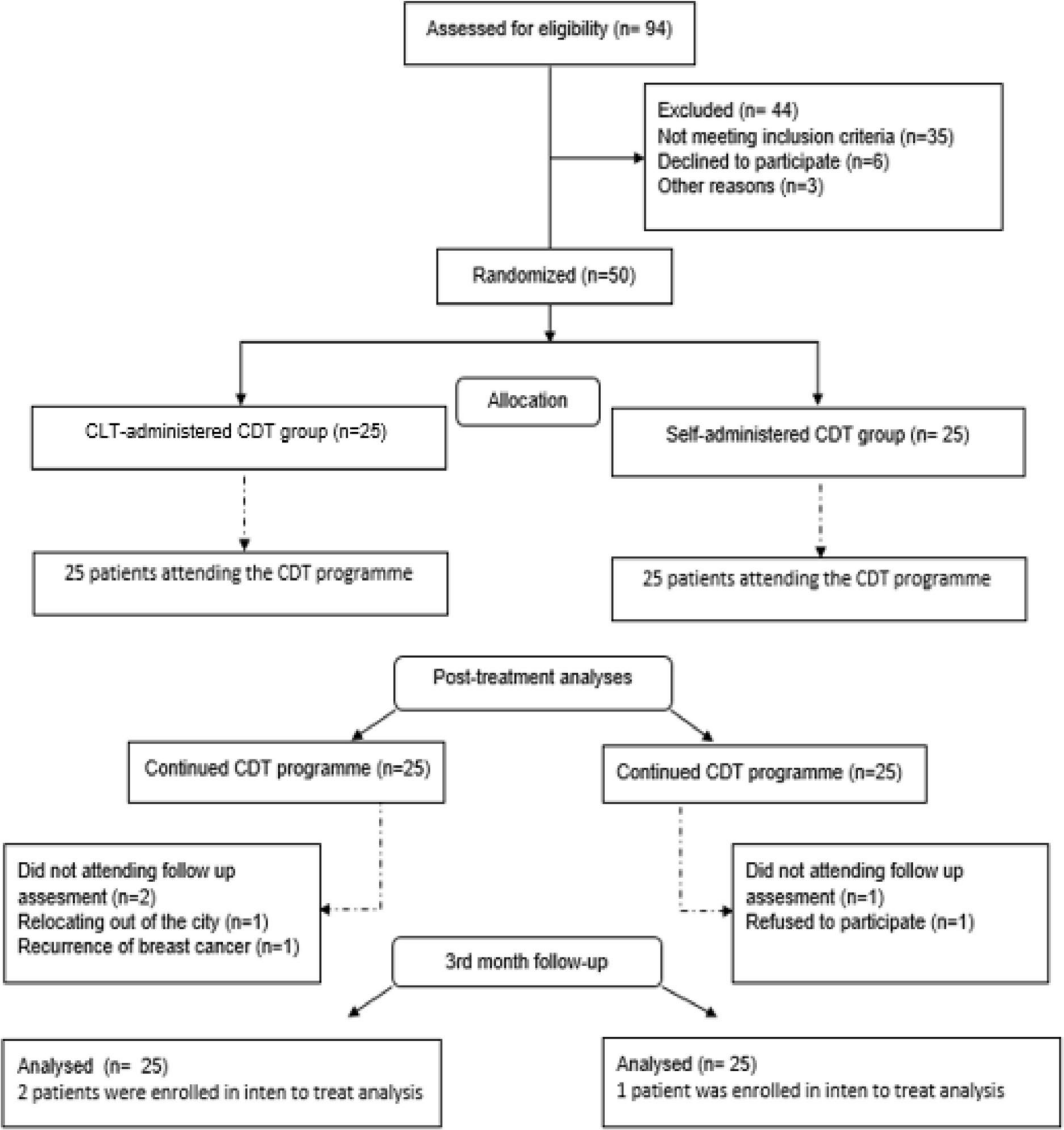
Flowchart of the study protocol

A total of 94 patients were evaluated for eligibility and 50 patients who met the inclusion criteria were enrolled in the study. The flowchart of the present study is presented in Fig. 1. The study was completed with 25 women with CLT-administered CDT group (age: 64.16 ± 10.02 years, BMI: 29.91 ± 5.38 kg/m2) and 25 self-administered CDT group (age: 62.28 ± 8.71 years, BMI: 31.25 ± 6.19 kg/m2) (Table 1). No between-group differences were detected in baseline assessments (*p* > 0.05, Table 1). The mean attendance for the 15-session CDT programme was 14.40 (SD = 1.04) in the CLT-administered CDT group and 14.48 (SD = 1.08) in the self-administered CDT group, yielding compliance rates of 96% for both groups (*p* > 0.05). At the 3-month follow-up, intention-to-treat analysis was performed to include the results of three patients (two in the CLT-administered group and one in the self-administered group). Arm sleeve use was 80% in the CLT-administered group and 68% in the self-administered group (*p* > 0.05). Among the self-administered group, 70.83% reapplied the CDT programme, primarily due to factors like arm sleeve incompatibility (33.44%) or other reasons (66.56%). Adherence to the 15-session walking recommendation was 75.46% (mean = 11.32 sessions, SD = 2.32) in the CLT-administered group and 80.53% (mean = 12.08 sessions, SD = 2.34) in the self-administered group, with no significant difference between groups (*p* > 0.05).

**Table 1:** Baseline characteristics of groups

	CLT-CDT group (*n* = 25)	Self-CDT group (*n* = 25)	*p*-value
	Mean ± SD, Median (IQR 25/75) or n (%)		
Demographic Characteristics			
Age (years)	64.16 ± 10.02	62.28 ± 8.71	0.482^a^
Height (cm)	155.44 ± 6.33	158.64 ± 5.57	0.064^a^
Weight (kg)	72.00 ± 12.25	78.76 ± 17.91	0.126^a^
BMI (kg/m^2^)	29.91 ± 5.38	31.25 ± 6.19	0.419^a^
Marriage status (married)	17 (68%)	18 (72%)	0.758^c^
Working status (working)	3 (12%)	2 (8%)	0.637^d^
Affected extremity (Dominant)	13 (52%)	11 (44%)	0.57^c^
Chronic disease (yes)	15 (60%)	20 (80%)	0.123^c^
Family history of cancer (yes)	14 (56%)	16 (64%)	0.564^c^
Smoking (yes)	0 (0%)	1 (4%)	> 0.999^d^
History of CDT treatment	17 (68%)	15 (60%)	0.556^c^
Disease-Related Characteristics			
BC stage (AJCC)			
Stage I	3 (%12)	4 (%16)	0.687^d^
Stage II	14 (%56)	13 (%52)	0.777^c^
Stage III	8 (32%)	8 (32%)	> 0.999^d^
Histological subtype			
Invasive ductal carcinoma	21 (84%)	22 (88%)	0.687^d^
Invasive lobular carcinoma	3 (12%)	2 (8%)	0.641^d^
Other	1 (4%)	1 (4%)	> 0.999^d^
BC-free survival time (months)	188.00 (83.75–242.00)	52.00 (27.25–169.00)	0.107^b^
Extremity of volume differences (cm^3^)	1151.70 ± 486.57	1270.67 ± 586.62	0.439^a^
ISL lymphedema stage (severe)	17 (68%)	16 (64%)	0.556^c^
Duration of lymphedema (months)	72.00 (23.50–122)	52.00 (27.25–169.00)	0.614^b^
Symptoms			
Stiffness (NRS: 0–10)	6.36 ± 1.52	7.28 ± 2.01	0.075^a^
Pain (NRS: 0–10)	2.84 ± 1.46	3.28 ± 1.51	0.301^a^
Heaviness (NRS: 0–10)	6.88 ± 2.33	7.04 ± 2.18	0.804^a^
Tingling (NRS: 0–10)	3.04 ± 1.71	2.68 ± 1.18	0.392^a^
Fatigue (NRS: 0–10)	6.84 ± 1.90	7.20 ± 1.93	0.511^a^
Treatment			
Mastectomy (yes) Lumpectomy (yes) Axillar dissection (yes)	21 (84%) 4 (16%) 25 (100%)	19 (76%) 6 (24%) 25 (100%)	0.480^d^ 0.713^d^ > 0.999^d^
RT (yes)	24 (96%)	21 (84%)	0.157^d^
WBRT	15 (60%)	14 (56%)	0.774^c^
RNI ± WBRT	9 (36%)	7 (28%)	0.544^c^
CT (yes)	25 (100%)	24 (96%)	0.312^d^
Range of Motion			
Shoulder flexion (^o^)	162.80 ± 12.83	161.20 ± 11.83	0.649^a^
Shoulder extension (^o^)	26.40 ± 6.69	27.80 ± 4.80	0.400^a^
Shoulder abduction (^o^)	147.80 ± 19.36	147.60 ± 14.21	0.967^a^
Shoulder adduction (^o^)	32.40 ± 4.35	33.20 ± 4.37	0.566^a^
Shoulder IR (^o^)	69.60 ± 12.40	70.76 ± 12.30	0.731^a^
Shoulder ER (^o^)	72.80 ± 12.75	69.60 ± 10.88	0.345^a^
Elbow flexion (^o^)	134.40 ± 6.97	137.40 ± 7.37	0.146^a^
Wrist flexion (^o^)	58.20 ± 12.57	54.00 ± 11.63	0.226^a^
Wrist extension (^o^)	53.00 ± 13.84	55.60 ± 10.13	0.453^a^
Peripheral Muscle Strength			
Hand grip strength (kg)	18.24 ± 4.56	18.16 ± 3.28	0.944^a^
UE Disability Level			
DASH (0–100)			
Disability	31.17 ± 10.76	33.67 ± 11.11	0.423^a^
Work	36.40 ± 17.93	34.10 ± 17.16	0.646^a^
Physical Activity Level			
IPAQ-SF (MET-min/week)	132.00 (00.00–297.00)	00.00 (00.00–222.75)	0.413^b^
Fatigue			
CIS (20–140)			
Subjective fatigue	28.16 ± 8.72	24.76 ± 8.55	0.171^a^
Concentration	20.56 ± 6.29	20.04 ± 5.90	0.908^a^
Motivation	15.68 ± 5.28	14.96 ± 5.97	0.654^a^
Activity	12.08 ± 4.20	11.96 ± 4.42	0.921^a^
Total Score	76.48 ± 11.63	71.52 ± 9.46	0.105^a^
HRQoL			
ULL-27 (0–100)			
Physical	58.86 ± 8.58	59.73 ± 9.67	0.873^a^
Physiologic	44.57 ± 2.75	38.57 ± 3.26	0.361^a^
Social	33.00 ± 8.92	29.60 ± 8.59	0.525^a^
Total score	50.37 ± 5.26	48.66 ± 5.20	0.694^a^

*SD* Standard deviation, *n* Number, *%* Percentage, *IQR 25/75* Interquartile range between 25 and 75th percentiles, *BMI* Body Mass Index, *cm*: Centimetre, *kg* Kilogram, *m* Metre, *CLT-CDT*: Certified lymphedema therapist-administered CDT, *Self-CDT* Self-administered *CDT, CDT* Complex decongestive therapy, *BC* Breast cancer, *AJCC* American Joint Committee on Cancer, *UE* Upper extremity, *ISL* The International Society of Lymphology, *CT* Chemotherapy, *RT* Radiotherapy, *WBRT* Whole breast radiation therapy, *RNI ± WBRT* Regional nodal irradiation with or without whole breast radiation therapy, *IR* Internal rotation, *ER* External rotation, *°* Degree, *DASH* Disabilities of the arm, shoulder and hand, *IPAQ-SF* International Physical Activity Questionnaire-Short Form, *CIS* checklist individual strength, *HRQoL* Health-related quality of life, *ULL-27* Upper Limb Lymphedema 27 Questionnaire

^a^Student’s t-test

^b^Mann–Whitney U-test

^c^Chi-square test

^d^Fisher’s exact test

^*^p < 0.05

### Within-group comparisons

**Fig. 2:**
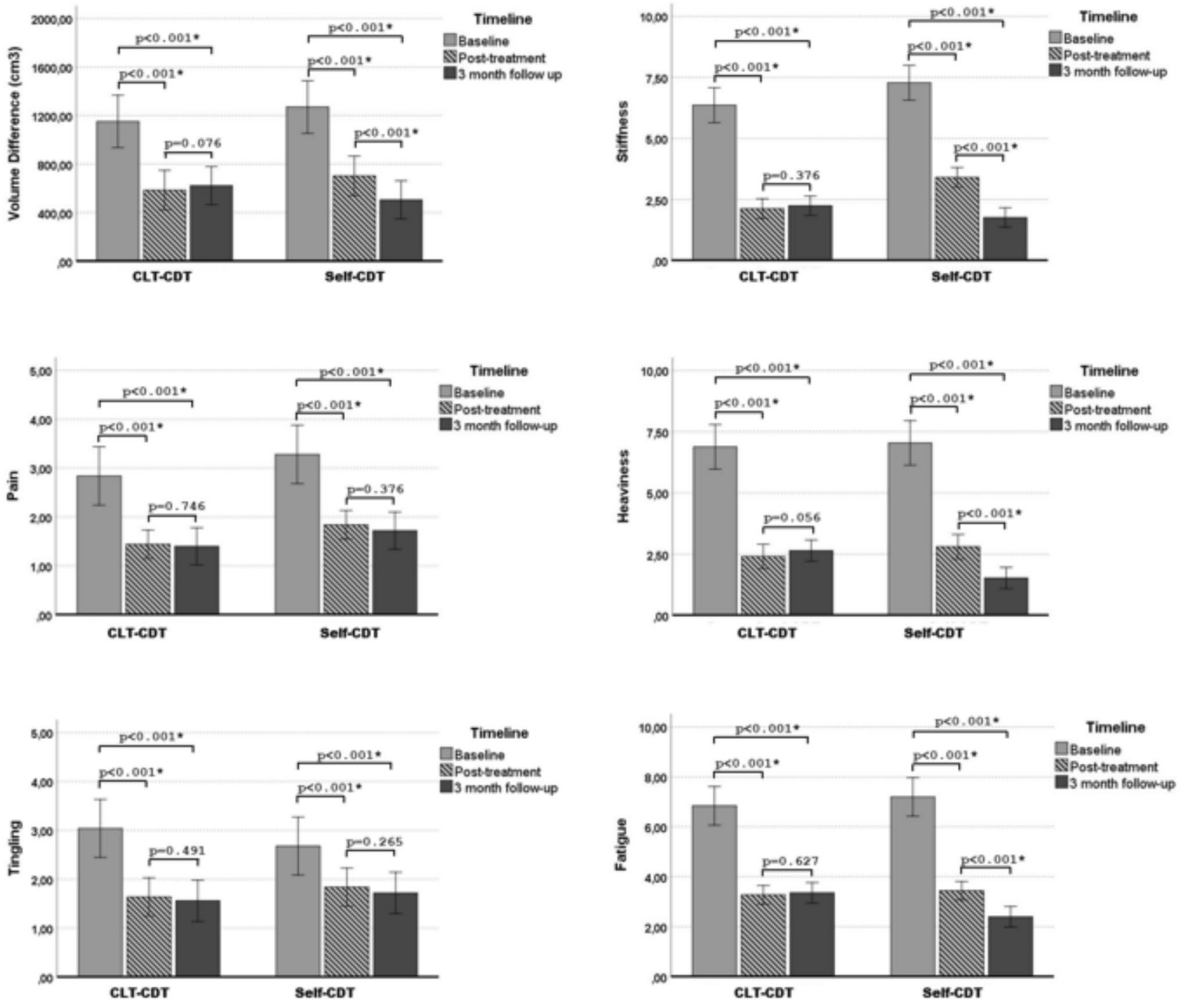
Change in Lymphedema Severity (cm^3^) and Lymphedema-Related Symptoms Across Study Time Points. Repeated-measures ANOVA analyses revealed a time-dependent change in lymphedema severity (cm^3^) and lymphedema-related symptoms in both groups (*p *< 0.05). Error bars represent standard deviation. Bonferroni correction was applied to p values, with a significance threshold set at *p* < 0.017. CLT-CDT: Certified lymphedema therapist-administered complex decongestive therapy, *Self-CDT* Self-administered complex decongestive therapy

Following CDT, both the CLT-administered and self-administered groups showed significant reductions in lymphedema severity (*p* < 0.001; *d* = 1.24 and *d* = 0.93, respectively) and lymphedema-related symptoms (*p* < 0.001; *d* = 0.97–2.07; *d* = 0.75–2.12, respectively). The self-administered group also demonstrated significant improvements in ROMs (*p* < 0.05; *d* = 0.39–0.97), while the CLT-administered group showed improvements in all ROMs except shoulder adduction and external rotation (*p* > 0.05). Both groups had significant increases in hand grip strength (*p* < 0.001; *d* = 0.36 and *d* = 0.39, respectively) post-treatment. Additionally, both groups showed significant decreases in DASH disability/symptom and work module scores (*p* < 0.001; *d* = 1.34–1.83) and significant improvements in IPAQ-SF scores (*p* < 0.001; *d* = 3.10–3.65). The CLT-administered and self-administered CDT groups also experienced significant reductions in CIS total scores (*p* < 0.001; *d* = 1.51 and *d* = 1.98) and ULL-27 scores (*p* < 0.001; *d* = 2.68 and *d* = 2.57), including subparameters (*p* < 0.05; *d* = 0.30–2.69). The changes in the within-group lymphedema severity and health-related outcomes after CDT are summarized in Table 2. Repeated-measures ANOVA revealed a time-dependent change in lymphedema severity (cm^3^) and lymphedema-related symptoms in both groups (*p* < 0.05). The results of post hoc analyses showed that lymphedema severity (cm^3^) and lymphedema-related symptoms decreased in both groups after treatment and at 3-month follow-up compared to baseline measurements (*p* < 0.05). The severity of lymphedema (cm^3^) and lymphedema-related symptoms was maintained in the CLT-administered CDT group during the 3-month follow-up period (*p* > 0.05). Self-administered CDT group showed a time-dependent decrease in lymphedema severity (cm^3^), stiffness, heaviness and fatigue (*p* < 0.05), while pain and tingling were preserved after treatment (*p* > 0.05). Post hoc analysis results are shown in Fig. 2.

**Table 2:** Within-group comparisons of baseline and post-treatment outcomes

	CLT-CDT group Mean ± SD, Median (IQR 25–75)				Self-CDT group Mean ± SD, Median (IQR 25–75)			
	Baseline	Post-treatment	p-value	ES	Baseline	Post-treatment	*p*-value	ES
Lymphedema Severity								
Extremity of volume Differences (cm^3^)	1151.70 ± 486.57	584.64 ± 361.52	** < 0.001**^a,*^	1.24†	1270.67 ± 586.62	702.43 ± 445.27	** < 0.001**^a,*^	0.93†
Extremity of volume Differences (%)	40.95 (33.84–60.78)	19.92 (11.85–39.31)	** < 0.001**^b,*^	3.59†	54.02 (44.48–75.41)	26.34 (19.03–47.56)	** < 0.001**^b,*^	3.28†
Symptoms								
Stiffness (NRS: 0–10)	6.36 ± 1.52	2.12 ± 0.97	** < 0.001**^a,*^	1.54†	7.28 ± 2.01	3.40 ± 1.04	** < 0.001**^a*^	2.12†
Pain (NRS: 0–10)	2.84 ± 1.46	1.44 ± 0.58	** < 0.001**^a,*^	1.21†	3.28 ± 1.51	1.84 ± 0.85	** < 0.001**^a*^	1.06†
Heaviness (NRS: 0–10)	6.88 ± 2.33	2.40 ± 1.22	** < 0.001**^a,*^	1.56†	7.04 ± 2.18	2.80 ± 1.25	** < 0.001**^a,*^	1.78†
Tingling (NRS: 0–10)	3.04 ± 1.71	1.64 ± 0.90	** < 0.001**^a,*^	0.97†	2.68 ± 1.18	1.84 ± 1.02	** < 0.001**^a,*^	0.75†
Fatigue (NRS: 0–10)	6.84 ± 1.90	3.28 ± 0.84	** < 0.001**^a,*^	2.07†	7.20 ± 1.93	3.44 ± 1.00	** < 0.001**^a,*^	1.92†
Range of Motion								
Shoulder flexion (^o^)	162.80 ± 12.83	170.00 ± 9.57	** < 0.001**^a,*^	0.59†	161.20 ± 11.83	169.80 ± 9.40	** < 0.001**^a,*^	0.73†
Shoulder extension (^o^)	26.40 ± 6.69	28.56 ± 7.68	**0.010**^a*^	0.52†	27.80 ± 4.80	30.00 ± 7.07	**0.024**^a,*^	0.55†
Shoulder abduction (^o^)	147.80 ± 19.36	159.00 ± 16.77	** < 0.001**^a,*^	0.60†	147.60 ± 14.21	160.80 ± 12.63	** < 0.001**^a,*^	0.97†
Shoulder adduction (^o^)	32.40 ± 4.35	32.80 ± 4.10	0.161^a^	0.09§	33.20 ± 4.37	36.80 ± 9.66	**0.044**^a,*^	0.43‡
Shoulder IR (^o^)	69.60 ± 12.40	73.84 ± 10.50	**0.014**^a*^	0.36‡	70.76 ± 12.30	74.60 ± 9.11	**0.011**^a*^	0.35‡
Shoulder ER (^o^)	72.80 ± 12.75	76.20 ± 9.71	0.153^a^	0.28§	69.60 ± 10.88	72.20 ± 10.61	**0.012**^a,*^	0.41‡
Elbow flexion (^o^)	134.40 ± 6.97	141.76 ± 7.01	** < 0.001**^a,*^	1.05†	137.40 ± 7.37	141.60 ± 4.72	**0.001**^a,*^	0.62†
Wrist flexion (^o^)	58.20 ± 12.57	64.20 ± 9.20	**0.002**^a*^	0.49‡	54.00 ± 11.63	59.72 ± 10.28	** < 0.001**^a,*^	0.44‡
Wrist extension (^o^)	53.00 ± 13.84	58.00 ± 12.24	**0.001**^a*^	0.37‡	55.60 ± 10.13	61.00 ± 10.10	** < 0.001**^a,*^	0.41‡
Peripheral Muscle Strength								
Hand grip strength (kg)	18.24 ± 4.56	19.80 ± 4.02	** < 0.001**^a,*^	0.36‡	18.16 ± 3.28	19.44 ± 2.91	** < 0.001**^a,*^	0.39‡
UE Disability Level								
DASH (0–100)								
Disability	31.17 ± 10.76	17.70 ± 7.93	** < 0.001**^a,*^	1.34†	33.67 ± 11.11	15.09 ± 6.32	** < 0.001**^a,*^	1.83†
Work	36.40 ± 17.93	16.25 ± 7.22	** < 0.001**^a,*^	1.19†	34.10 ± 17.16	15.61 ± 8.24	**0.014**^a,*^	1.23†
Physical Activity Level								
IPAQ-SF (MET-min/week)	132.00 (00.00–297.00)	495.00 (346.50–717.75)	** < 0.001**^b*^	3.65†	00.00 (00.00–222.75)	445.50 (247.50–495.00)	** < 0.001**^b,*^	3.10†
Fatigue								
CIS (20–140)								
Subjective fatigue	28.16 ± 8.72	23.00 ± 4.78	**0.019**^a*^	0.30‡	24.76 ± 8.55	18.48 ± 3.54	**0.002**^a,*^	0.88†
Concentration	20.56 ± 6.29	15.84 ± 3.00	**0.003**^a*^	0.40‡	20.04 ± 5.90	14.56 ± 1.68	** < 0.001**^a,*^	0.92†
Motivation	15.68 ± 5.28	10.04 ± 3.72	** < 0.001**^a,*^	0.93†	14.96 ± 5.97	10.88 ± 3.21	**0.002**^a,*^	1.42†
Activity	12.08 ± 4.20	8.56 ± 2.91	**0.001**^a,*^	0.97†	11.96 ± 4.42	9.72 ± 3.15	**0.037**^a,*^	0.59†
Total score	76.48 ± 11.63	57.44 ± 8.78	** < 0.001**^a,*^	1.51†	71.52 ± 9.46	53.64 ± 8.51	** < 0.001**^a,*^	1.98†
HRQoL								
ULL-27 (0–100)								
Physical	58.86 ± 8.58	13.93 ± 9.59	** < 0.001**^**a**^	2.69†	59.73 ± 9.67	13.60 ± 5.84	** < 0.001**^a*^	2.56†
Physiologic	44.57 ± 2.75	15.71 ± 1.19	** < 0.001**^**a**^	1.42†	38.57 ± 3.26	15.85 ± 1.25	** < 0.001**^a,*^	1.11†
Social	33.00 ± 8.92	6.00 ± 5.14	** < 0.001**^**a**^	1.46†	29.60 ± 8.59	6.20 ± 3.64	** < 0.001**^a,*^	1.46†
Total score	50.37 ± 5.26	13.03 ± 6.68	** < 0.001**^**a**^	2.68†	48.66 ± 5.20	12.81 ± 5.02	** < 0.001**^a,*^	2.57†

The expression [bold] represents statistical significance (*p* < 0.05)

*SD* Standard deviation, *n* Number, *%* Percentage, *IQR 25/75*, Interquartile range between 25 and 75th percentiles, *CLT-CDT* Certified lymphedema therapist-administered CDT, *Self-CDT* Self-administered CDT, *CDT*: Complex decongestive theraphy, *UE* Upper extremity, *NRS* Numeric rating scale, *IR* Internal rotation, *ER* External rotation *°* Degree, *DASH* Disabilities of the arm, shoulder and hand, *IPAQ-SF* International Physical Activity Questionnaire-Short Form, *CIS* Checklist individual strength, *HRQoL* Health-related quality of life, *ULL-27* Upper Limb Lymphedema 27 Questionnaire

^a^Student’s *t*-test

^b^Mann–Whitney U-test

^*^p < 0.05, ES: Effect size,

^†^large effect size (*d* = 0.50–1.0),

^‡^moderate effect size (*d* = 0.30–0.49),

^§^small effect size (*d* = 0.10–0.20)

### Between-group comparisons

No significant difference was observed between the groups in the mean change following CDT for lymphedema severity (*p* = 0.988; *d* = 0.04) and lymphedema-related symptoms (*p* > 0.05; *d* = 0.02–0.41). However, at the 3-month follow-up, there was a significant difference in lymphedema severity (*p* < 0.001; *d* = 1.40) and symptoms of stiffness, heaviness and fatigue (*p* < 0.001; *d* = 2.09–1.27). There were no significant between-group differences in pain (*p* = 0.660; *d* = 0.12) or tingling (*p* = 0.798; *d* = 0.07) at the 3-month follow-up. Comparisons between groups showed no statistically significant differences in ROMs changes (*p* > 0.05; *d* = 0.09–0.55), hand grip strength (*p* = 0.415; *d* = 0.23), DASH/disability (*p* = 0.019; *d* = 0.55), DASH/work (*p* = 0.694; *d* = 0.11), IPAQ-SF scores (*p* = 0.296; *d* = 0.44) or CIS total and subscores (*p* > 0.05; *d* = 0.08–0.26). No significant between-group differences were found in the PBI (*p* = 0.403; *d* = 0.23) and ULL-27 total and subscores (*p* > 0.05; *d* = 0.11–0.35). The changes in the mean differences between the groups are shown in Table 3.

**Table 3:** Comparison of changes at post-treatment and 3rd-month follow-up between groups

	CLT-CDT group	Self-CDT group	*p*-value	ES
	Mean Differences (95% CI)			
Lymphedema Severity				
Δ Volume differences (cm^3^)	−567.06 (−439.65/−694.46)	−568.24 (−472.24/−664.23)	0.988^a^	0.04§
Δ Volume differences (cm^3^) at 3rd mo	37.42 (17.51/57.32)	−197.56 (−281.05/−114.06)	** < 0.001**^a*^	1.40†
Δ Volume differences (%)	−23.72 (−28.60/−18.84)	−27.71 (−32.55/−22.86)	0.262^a^	0.32‡
Δ Volume differences (%) at 3rd mo	2.16 (−0.07/4.39)	−8.78 (−12.76/−4.80)	** < 0.001**^a*^	1.34†
Symptoms				
Δ Stiffness (NRS: 0–10)	−4.24 (−4.66/−3.81)	−3.88(−4.49/−3.26)	0.335^a^	0.26§
Δ Stiffness at 3rd mo (NRS: 0–10)	0.12 (−0.13/0.37)	−1.64 (−2.04/−1.23)	** < 0.001**^a*^	2.09†
Δ Pain (NRS: 0–10)	−1.40 (−1.97/−0.82)	−1.44 (−1.91/−0.96)	0.917^a^	0.02§
Δ Pain at 3rd mo (NRS: 0–10)	−0.40 (−0.67/−0.13)	−0.12 (−0.37/−0.13)	0.660^a^	0.12§
Δ Heaviness (NRS: 0–10)	−4.48 (−5.13/−3.82)	−4.24 (−4.75/−3.73)	0.572^a^	0.16§
Δ Heaviness at 3rd mo (NRS: 0–10)	0.24 (0.47/−0.05)	−1.28 (−1.68/−0.88)	** < 0.001**^a*^	1.81†
Δ Tingling (NRS: 0–10)	−1.40 (−2.04/−0.75)	− 0.84 (−1.21/−0.46)	0.153^a^	0.41‡
Δ Tingling at 3rd mo (NRS: 0–10)	−0.80 (−1.02/−0.57)	−0.13 (−0.33/−0.07)	0.798^a^	0.07§
Δ Fatigue(NRS: 0–10)	−3.56 (−4.17/−2.94)	−3.76 (−4.28/−3.23)	0.631^a^	0.13§
Δ Fatigue at 3rd mo (NRS: 0–10)	0.80 (0.48/1.11)	−1.04 (−1.405/−0.67)	** < 0.001**^a*^	1.27†
Range of Motion				
Δ Shoulder flexion (^o^)	7.20 (5.46/8.93)	8.60 (6.89/10.30)	0.55^a^	0.16§
Δ Shoulder extension (^o^)	2.16 (0.63/3.68)	2.20 (0.40/3.99)	0.974^a^	0.09§
Δ Shoulder abduction (^o^)	11.20 (10.69/11.70)	13.20 (11.89/14.50)	0.466^a^	0.20§
Δ Shoulder adduction (^o^)	4.40 (3.85/4.94)	4.60 (3.62/4.57)	0.069^a^	0.52†
Δ Shoulder IR (^o^)	4.24 (1.12/7.35)	3.84 (1.11/6.56)	0.851^a^	0.55†
Δ Shoulder ER (^o^)	4.20 (3.71/ 4.68)	2.60 (1.49/3.70)	0.516^a^	0.18§
Δ Elbow flexion (^o^)	7.36 (4.08/10.63)	4.20 (2.03/6.36)	0.121^a^	0.44‡
Δ Wrist flexion (^o^)	6.00 (2.65/9.34)	5.72 (3.16/8.27)	0.897^a^	0.37‡
Δ Wrist extension (^o^)	5.00 (2.28/7.71)	5.40 (3.53/7.26)	0.813^a^	0.55†
Peripheral Muscle Strength				
Δ Hand grip strength (kg)	1.64 (0.97/2.30)	1.28 (0.74/1.81)	0.415^a^	0.23§
UE Disability Level				
DASH (0–100)				
Δ Disability	−13.47 (−15.94/−10.99)	−18.57 (−20.87/−14.26)	0.019^a^	0.55†
Δ Work	−20.14 (−25.84/−14.43)	−18.49 (−24.37/−12.61)	0.694^a^	0.11§
Physical Activity Level				
Δ IPAQ-SF (MET-min/week)	369.60 (300.57/438.63)	313.50 (235.70/391.29)	0.296^a^	0.44‡
Fatigue				
CIS (20–140)				
Δ Subjective fatigue	−5.16 (−9.19/−1.12)	−6.00 (−9.46/−2.53)	0.758^a^	0.08§
Δ Concentration	−4.72 (−7.53/−1.90)	−5.48 (−8.10/−2.85)	0.751^a^	0.10§
Δ Motivation	−5.64 (−8.11/−3.16)	−4.08 (−6.37/−1.78)	0.370^a^	0.25§
Δ Activity	−3.52 (−5.38/−1.65)	−2.24 (−4.22/−0.25)	0.362^a^	0.26§
Δ Total score	−19.04 (−25.14/−12.93)	−17.78 (−21.46/−14.09)	0.751^a^	0.09§
Treatment Benefits and Goals				
PBI−Lymphedema (0–4)				
Δ First subdimension	3.08 (2.90/3.25)	3.08 (2.91/3.24)	0.998^a^	0.001§
Δ Second subdimension	2.11(1.80/2.41)	2.34 (2.06/2.61)	0.286^a^	0.30‡
Δ Total score	2.69 (2.50/2.87)	2.80 (2.62/2.97)	0.403^a^	0.23§
HRQoL				
ULL-27 (0–100)				
Δ Physical	−44.93 (−50.79/−39.06)	−46.13 (−52.88/−39.37)	0.794^a^	0.35‡
Δ Psychological	−28.85 (−36.23/−21.46)	−22.71 (−30.47/−14.94)	0.267^a^	0.31‡
Δ Social	−26.40 (−32.86/−19.94)	−23.40 (−30.01/−16.78)	0.528^a^	0.18§
Δ Total score	−37.33 (−42.20/−32.45)	−35.85 (−40.97/−30.72)	0.683^a^	0.11§

The expression [bold] represents statistical significance (*p* < 0.05)

*SD* Standard deviation, *n* Number, *%* Percentage, *IQR 25/75* Interquartile range between 25 and 75th percentiles, *CLT-CDT* Certified lymphedema therapist-administered CDT, *Self-CDT* Self-administered CDT, *CDT* Complex decongestive theraphy, *3rd mo* Difference of change after treatment and 3 months, *UE* Upper extremity, *IR* Internal rotation, *ER* External rotation, *NRS* Numeric rating scale, *°* Degree, *DASH* Disabilities of the arm, shoulder and hand, *IPAQ-SF* International Physical Activity Questionnaire-Short Form, *CIS* Checklist individual strength, *PBI* Patient benefit index, *HRQoL* Health-related quality of life, *ULL-27* Upper Limb Lymphedema 27 Questionnaire

^a^Student’s t-test

^*^p < 0.05, ES: Effect size

^†^large effect size (*d* = 0.50–1.0)

^‡^moderate effect size (*d* = 0.30–0.49)

^§^small effect size (*d* = 0.10–0.29)

Δ: Difference between after treatment and baseline outcomes

Δ at 3rd mo: Difference between the 3rd-month follow-up and after treatment outcomes

## Discussion

The main findings of the present study were as follows: (1) the effectiveness of self-administered CDT on lymphedema management and health-related outcomes is not inferior to the results yielded from CLT-administered CDT. (2) The 3-month follow-up revealed a slight increase in lymphedema severity after 15 sessions of CLT-administered CDT group without statistical significance. On the other hand, lymphedema severity continued to decrease in the self-administered CDT group. (3) Lymphedema-related symptoms decreased in both groups after 15 sessions of CDT and, remarkably, the reduction of symptoms in the self-administered CDT group was maintained during the 3-month follow-up period, and stiffness, heaviness and fatigue decreased even further during follow-up.

Following 15 sessions of CDT treatment, the percentage change in volume difference was 49.2% in the CLT-administered CDT group and a comparable value of 44.7% in the self-administered CDT groups. Previous studies have consistently shown CDT to result in significant reductions in extremity volume, generally 30–50% [6, 7], and the results obtained in both groups in the current study are similar to previous studies. Previous follow-up studies evaluating the sustainability of treatment efficacy after CDT reported a tendency for lymphedema severity to be maintained or slightly increased during the post-treatment period [31–33]. The current study, in line with previous studies, revealed a slight increase in lymphedema severity in the CLT-administered CDT group at the end of the 3-month follow-up period (no statistically significant increase), while in the self-administered CDT group, lymphedema severity continued to decrease following 15-session CDT. This continuing improvement may be explained by the reapplication of CDT by a significant proportion of patients in the self-administered group (70.83%) during the follow-up period. The current study found that self-administered CDT was as effective as CLT-administered CDT in reducing lymphedema severity following 15 sessions. Notably, during the 3-month follow-up, self-administered CDT showed greater efficacy, with a 197.56 ml reduction in severity, compared to a 37.42 ml increase in the CLT-administered group. This highlights the potential for long-term improvement with self-administered treatment. Given the lack of government reimbursement for lymphedema management in most countries and the high cost of care [34], self-administered CDT may be a cost-effective alternative. The self-administered group received just one 2-h training session and three 15-min follow-ups, while the CLT-administered group received over 15 h of therapist-led treatment. This difference is particularly relevant given the shortage of healthcare providers and the high treatment costs [4]. The follow-up outcomes of self-administered CDT showed promising results in overcoming this barrier to the management of lymphedema in the present study. Encouraging patients to take control of their own care and educating patients on self-administered CDT protocols may benefit patients in terms of both facilitating access to treatment and reducing costs [14, 34].

Lymphedema often causes physical symptoms such as pain, heaviness, tingling, and so on, affecting the participation in daily activities [3, 22]. Previous studies have demonstrated the efficacy of CDT in alleviating these symptoms [9, 10]. Consistently in the present study, both groups had reduced lymphedema-related symptoms after 15 CDT sessions. However, the notable finding was the stabilization of symptoms in the CLT-administered CDT group during Phase II, while the self-administered CDT group showed sustained improvements, particularly in stiffness, heaviness and fatigue. This highlights the critical role of self-care, as not only did the self-administered CDT alleviate symptoms, but patients also achieved superior results to CLT-administered CDT in independently managing their condition.

ROMs, peripheral muscle strength and DASH are commonly used methods to assess upper extremity functionality in patients with BCRL [35]. Previous findings demonstrated a therapeutic effect of CDT on upper extremity ROMs [7]. The current study also observed improvements in ROMs in both groups following CDT. On the other hand, previous studies investigating self-care interventions primarily focused on usual care (using an arm sleeve, exercise and skin care) or self-MLD and did not report significant improvements in ROM [21]. The integration of self-applied bandaging into self-care in the present study seems to be the leading factor for this favourable result. Hand grip strength has been identified as a predictor of HRQoL and an increase of 1 kg has been associated with improved health outcomes [36]. The increases obtained from both self-administered CDT (1.28 kg) and CLT-administered CDT (1.64 kg) in the present study were above the cut-off value of 1 kg. This indicates CDT may be as effective as CLT-administered CDT in improving health outcomes, even in self-administered interventions. Previous research has demonstrated CLT-administered CDT results in an average change in DASH scores ranging from -8.60 to -18.33 [37, 38]. The self-administered CDT group achieved a mean difference of -18.49, which is comparable to the change observed with the professional-delivered intervention in the present study. Furthermore, there was no significant difference in mean DASH scores between the groups, indicating self-administered CDT effectively reduces upper limb disability to the same extent as CLT-administered CDT. The number of studies investigating the effect of self-care on upper extremity function in patients with BCRL is limited [35]. Additionally, a systematic review highlighted that even the best practice guidelines for the management of BCRL still rely on therapist-delivered interventions [7]. This study provides a comprehensive evaluation of self-administered CDT on upper extremity functionality and indicates it is non-inferior to CLT-administered CDT in improving upper extremity function.

The American Institute for Cancer Research (AICR) recommend cancer patients to engage in moderate-intensity exercises at least twice a week for at least 150 min [39]. However, most BCRL patients perform low levels of physical activity and do not meet the recommendations [40]. Similarly, the physical activity levels of patients in the current study were below this recommendation at the baseline. Considering lymphatic drainage is provided by musculoskeletal activation, the low physical activity levels of patients may have contributed to the progression of lymphedema severity [40]. Therefore, walking was recommended to all patients included in present study to help develop physical activity habits. Significant increases in physical activity levels were observed in both groups. However, the lack of follow-up of the increase in physical activity of the participants in the study makes it difficult to interpret the sustainability of this outcome.

The current study found a decrease in fatigue levels after CDT, particularly significant improvements in the concentration and motivation subscales in both groups. The possible reason for this may be attributed to the increase in physical activity level and the reduction in physical symptoms. However, the mean CIS scores of patients in both groups were still above the reported cut-off score of 35 following the interventions [41]. These findings highlight the need for additional approaches alongside CDT to further reduce fatigue in BCRL patients.

Previous studies have reported a mean total PBI-L score of 2.25 ± 0.71 following physiotherapist-delivered lymphedema treatment [28]. This study found similar results in both groups, suggesting patients were equally satisfied with both self-administered and CLT-administered CDT. Patient expectations play an important role in the development and sustainability of treatment plans as long-term compliance is more achievable when patients are satisfied with the treatment and perceive its benefits. The high level of adherence to the self-administered CDT (96.53%) and the high percentage of patients who reapplied treatment if necessary (70.83%) in the present study may be interpreted as resulting from positive patient satisfaction.

Considering the physical symptoms experienced by patients with lymphedema as well as the presence of cosmetic concerns, the HRQoL is an important issue in this population [29]. CDT is widely recognized as an effective conservative treatment for improving HRQoL in BCRL patients [42]. Previous research has demonstrated limited effects of self-administered MLD [6], and arm sleeves on improving HRQoL [43]. On the other hand, compared to other self-care protocols that included only certain aspects of CLT-administered CDT, the self-administered CDT protocol in the present study employed a more comprehensive multimodal approach, incorporating multi-layer bandaging for the first time. Our findings suggest that self-administered CDT leads to HRQoL improvements that other self-care programmes were not able to achieve. This highlights its potential as an effective, patient-centered treatment option for improving HRQoL in BCRL patients.

A strength of the present study is the comparison of the efficacy of the self-CDT protocol with CLT-administered CDT, which is considered the gold standard in the management of lymphedema. Additionally, the efficacy of self-administered CDT on functional and general health outcomes was evaluated using a comprehensive and multidimensional approach. The previous clinical trials for the management of lymphedema have included patients with moderate to severe lymphedema [7, 44]. The data of the current study show homogeneous distribution with the values existing literature and support the external validity of our results. This study has some limitations. Firstly, the intermediate follow-up period (3 months) reduces the long-term interpretability of the outcomes. Secondly, the 3-month follow-up period assessed only the primary outcome measures and did not analyse the effectiveness of self-CDT on functional status and other health-related outcomes of the patients.

## Conclusion

Prevention of disability from lymphedema is one of the key focus of BC survivorship care. Previous studies highlighted the importance of early intervention and self-care in the management of lymphedema [16, 34]. Moreover, given the chronic nature of the disease, self-care protocols potentially alleviate future burden [11, 12]. On the other hand, studies evaluating the efficacy of lymphedema self-care education indicate self-protocols (self-MLD, exercise, using arm sleeve or its combination) are insufficient to achieve the results obtained from CLT-administered CDT in lymphedema management [15, 17, 21]. However, the present study reveals that self-administered CDT protocol is not inferior to CLT-administered CDT in the management of lymphedema and reduction of lymphedema-related disabilities. Moreover, remarkably, the 3-month follow-up outcomes of self-administered CDT patients were also more successful than CLT-administered CDT in terms of control of lymphedema severity and related symptoms. These findings emphasize the potential for self-care to be as effective as physiotherapist-delivered treatment in the management of lymphedema. Encouraging patients to participate in self-CDT programmes may contribute to the sustainability of the healthcare system, considering lymphedema is a non-curative disease and the lack of healthcare professionals specialized in lymphedema. Additionally, self-administered CDT may be considered as a routine option for asymptomatic (subclinical stage) lymphedema in high-risk BC patients. However, future studies with larger sample sizes and longer follow-up may be beneficial to better understand the impact of self-administered CDT on lymphedema management and functional outcomes.

## Supplementary Information

Below is the link to the electronic supplementary material.

Supplementary file1 (PDF 891 kb)

Supplementary file2 (PDF 2886 kb)

## Data Availability

The datasets used and/or analysed during the present study are available from the corresponding author on reasonable request.
