# Supplementary material for: Self-administered versus lymphedema therapist-administered complex decongestive therapy protocol in breast cancer-related lymphedema: a non-inferiority randomized controlled trial with three-month follow-up
Source: Breast Cancer Res Treat. 2025 May 6;212(1):123–38. doi: 10.1007/s10549-025-07709-3 (PMC12086114; doi:10.1007/s10549-025-07709-3)
Supplement: Supplementary file 1 — Supplementary file1 (PDF 891 kb) [file 10549_2025_7709_MOESM1_ESM.pdf]

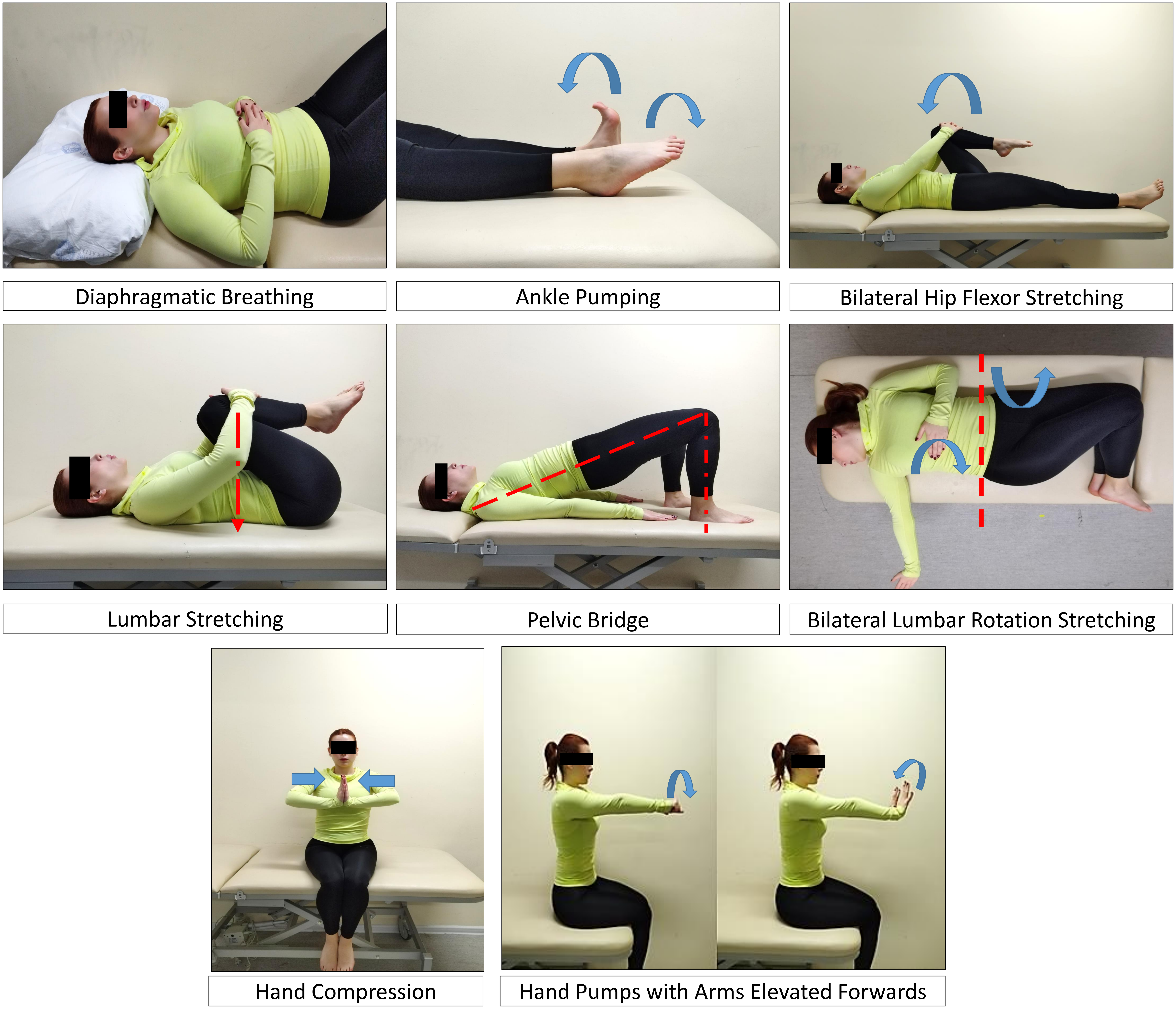
Diaphragmatic Breathing
Ankle Pumping
Bilateral Hip Flexor Stretching
Lumbar Stretching
Pelvic Bridge
Bilateral Lumbar Rotation Stretching
Hand Compression
Hand Pumps with Arms Elevated Forwards

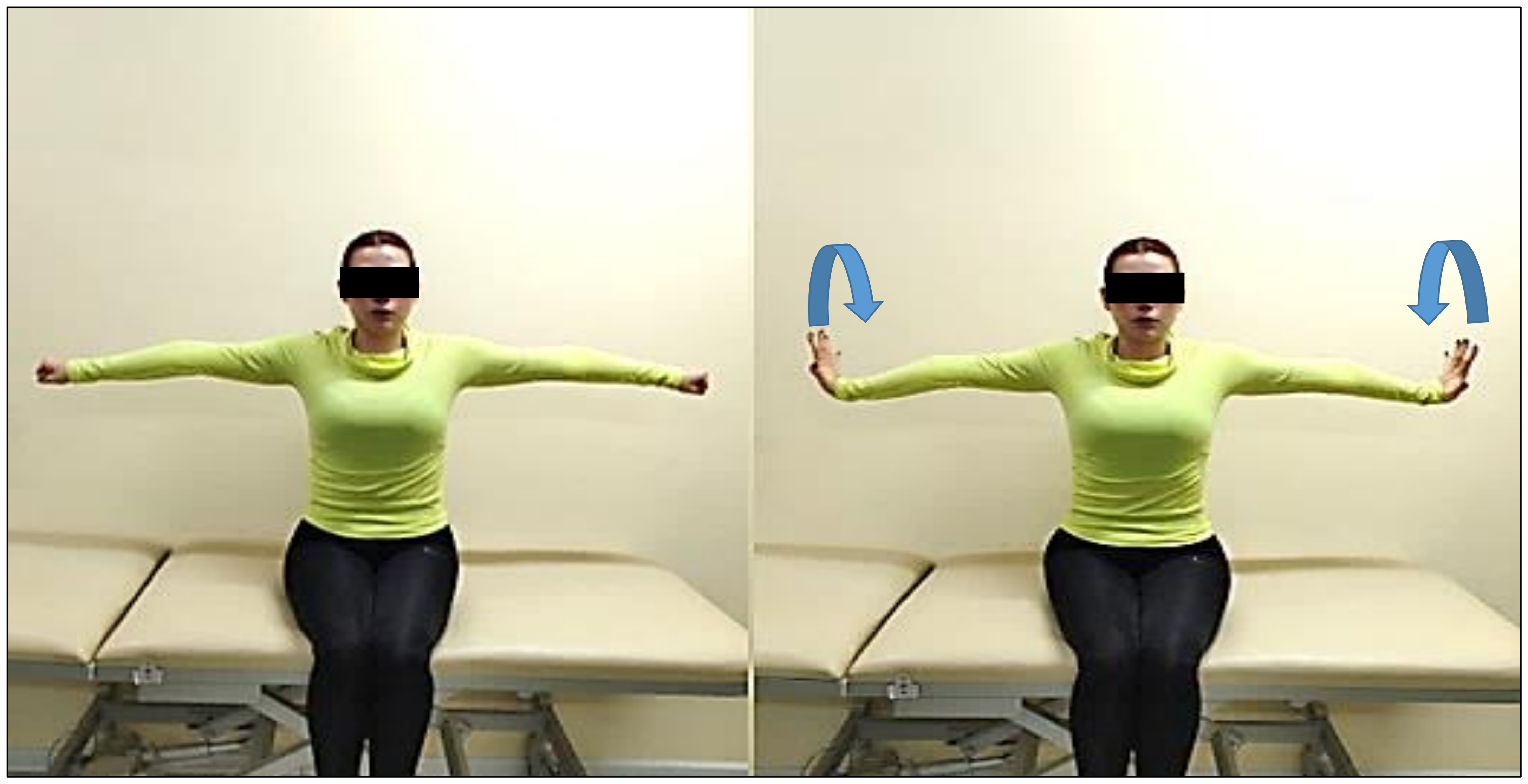

Hand Pumps with Arms Elevated to Side

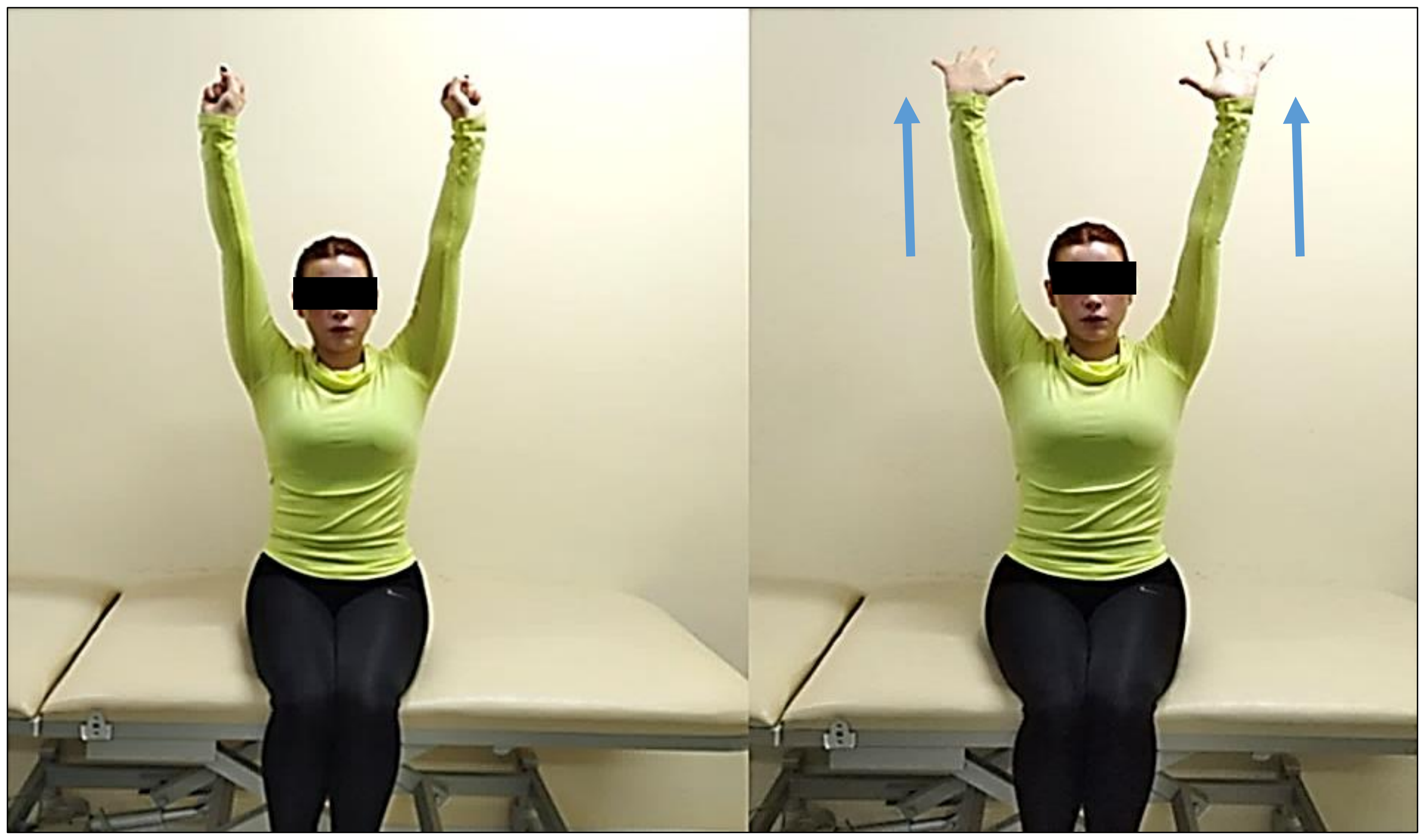

Hand Pumps with Arm Elevated Upwards

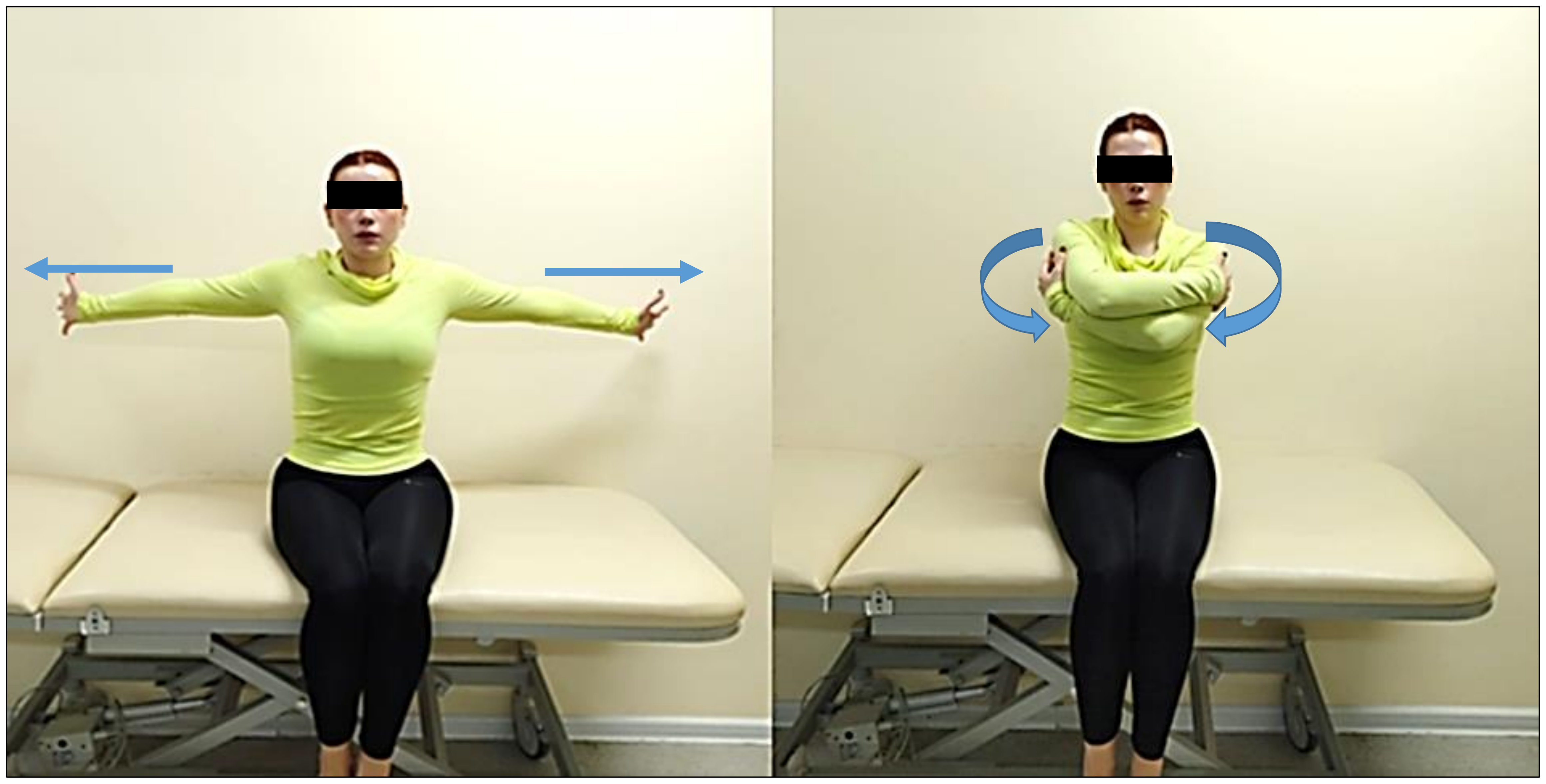

Arm Hug

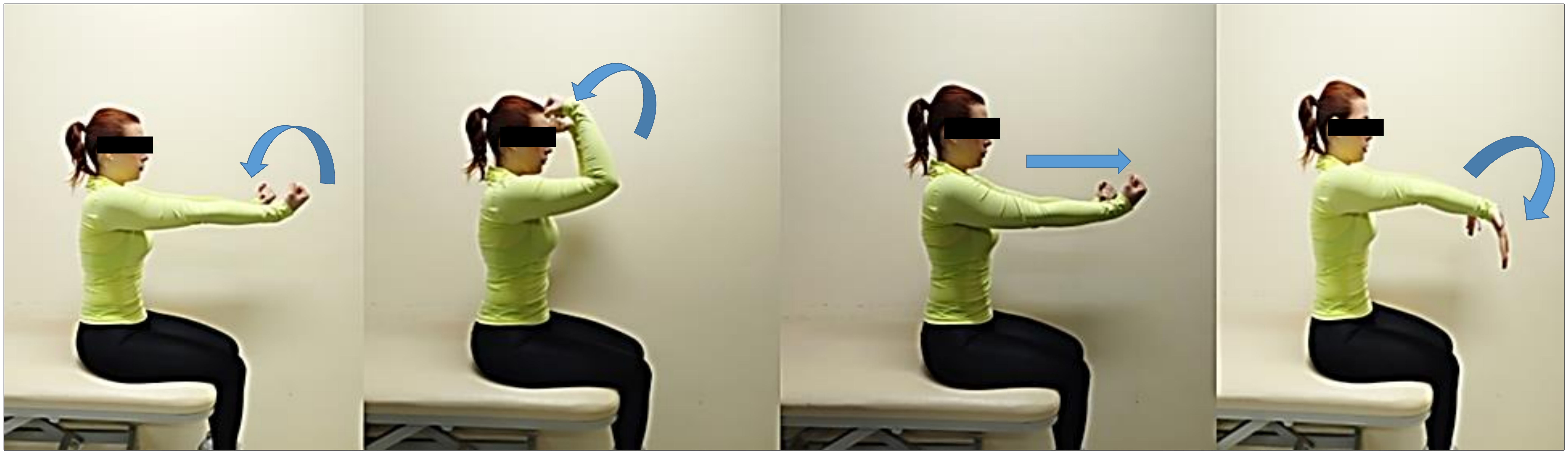

Dynamic Arm Stretching
