# Supplementary material for: Self-administered versus lymphedema therapist-administered complex decongestive therapy protocol in breast cancer-related lymphedema: a non-inferiority randomized controlled trial with three-month follow-up
Source: Breast Cancer Res Treat. 2025 May 6;212(1):123–38. doi: 10.1007/s10549-025-07709-3 (PMC12086114; doi:10.1007/s10549-025-07709-3)
Supplement: Supplementary file 2 — Supplementary file2 (PDF 2886 kb) [file 10549_2025_7709_MOESM2_ESM.pdf]

Contents

Chapter 1: Skin/ Nail Care 2

Chapter 2: Manual Lymph Drainage 5

Chapter 3: Multilayer Short Traction Bandage Application 14

Chapter 4: Exercises 22

Follow-up Diary 28

Chapter 1: Skin/ Nail Care

How Should I Care for My Skin/nails?

This chapter explains:

1. Why is skin/nail care important?
2. How to do self-skin/nail care step by step?

Warning: This booklet is personalized for you, do not recommend it to patients who have not had a pre-consultation with a lymphedema therapist.

Warning: Seek Medical Help if You Notice Signs of Infection

If you have lymphedema, it's important to stay alert for signs of infection. Lymphedema can make your skin more prone to infection, so if you notice any changes, seek medical attention right away.

Signs of Infection:

Redness or Warmth: The skin becomes red or feels hot to the touch.

Increased Swelling: Unusual or sudden swelling, especially if it's more intense than usual.

Pain or Tenderness: Pain or sensitivity in the affected area.

Fever or Chills: A rise in body temperature or chills along with skin changes.

Pus or Drainage: Any discharge from the skin, like pus or cloudy fluid.

Blisters or Open Sores: Painful blisters or sores that appear suddenly.

Tight or Shiny Skin: Skin that feels stretched, tight, or shiny.

Why You Need to Act Fast:

Infections can spread quickly and become serious. If you notice any of these signs, **don't wait**—get medical help right away to prevent the infection from worsening.

Prevention Tips:

Keep your skin clean and moisturized.

Avoid cuts and scratches.

Wear protective clothing when necessary.

Avoid pet bites, scratches

Beware of mosquito bites, use mosquito repellent spray if necessary

If you encounter one of these situations, apply a thin layer of antibiotic cream to the cut on the skin.

1. Why is skin/nail care important?

- Taking good care of your skin is crucial in preventing infections and other skin problems, such as fungal infections or dermatitis, especially when you have lymphedema.

- Lymphedema can damage the skin barrier of the affected area, making it more vulnerable to infection. Because of this, proper skin care should be a part of your daily routine, no matter what stage of treatment you're in. Consistently caring for your skin helps reduce the risk of complications and promotes overall health.

2. How to do self-skin/nail care step by step?

1. **Pay attention to hygiene recommendations:** If you have lymphedema, it’s essential to follow proper hygiene rules to prevent infections and damage to the skin barrier of the affected area. The natural pH of your skin is around 5, so it's recommended to clean your skin daily using low pH-balanced cleansers. These gentle cleansers help maintain the skin's natural acidic balance, preventing dryness and irritation.

2. **Use lukewarm water:** Hot water can dry your skin, so use lukewarm water when showering or washing your arm.

3. **Pat Dry:** To prevent moisture-related skin problems or fungal growth, you should not leave your skin damp. Gently pat your skin dry after every wash and shower. Remember to change your tea towel at regular intervals.

4. **Moisturize the skin:** Maintaining skin moisture is very important to prevent dryness or cracking. To do this, use low pH moisturizing creams or lotions daily.

5. **Wear Protective Clothing:** When outdoors or in situations where your skin may be exposed to dirt, UV rays, or other irritants, wear protective clothing to minimize risk.

6. **Avoid skin irritation:** Avoid epilation, scrubbing, peeling applications that can irritate the skin. If your skin itches, apply moisturizer and do not scratch with your nails.

7. **Nail cutting:** Cut your fingernails in a straight line. Do not cut your nails too short or with rounded edges. Avoid manicure applications.

Regularly Inspect Your Skin

Check your skin for redness, swelling, blisters, cuts or other changes.

Chapter 2: Manual Lymph Drainage

How Should I Perform Self-Manual Lymph Drainage for My Arm Lymphedema?

This chapter explains:

1) What is self-manual lymph drainage and why is it important?

2) How to perform self-manual lymph drainage step by step?

Warning: This booklet is personalized for you, do not recommend it to patients who have not had a pre-consultation with a lymphedema therapist.

1) What is self-manual lymph drainage and why is it important?

- Manual Lymph Drainage is a massage applied to the skin that helps move excess fluid from a swollen area to an area of your body where lymphatic circulation is healthy.

If you have swelling in your arm, trunk or chest area, direct the fluid to the area with intact lymph nodes. For example, if you develop swelling in your left arm, you should direct the fluid to the lymph nodes in your right armpit, the lymph nodes in your left neck and the lymph nodes in your left groin. Healthy lymph nodes, filter the diverted fluid and help return it to the heart. ***See Figure 1.***

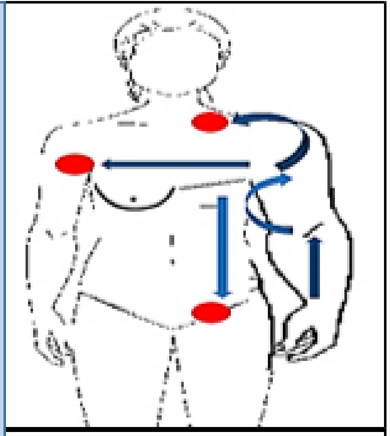

Figure 1

2) How to perform self-manual lymph drainage step by step?

Warning: It is important that this massage is gentle and slow.

1. Find a comfortable position: Sit or lie down in a quiet place away from distractions. Make sure your body is relaxed. Begin with diaphragmatic breathing, which helps to increase lymph flow. Repeat diaphragmatic breathing throughout the day as you remember it, doing five to seven repetitions.

2. Hand placement: Place your hand gently on your skin. Use the flat parts of your palms, not your fingertips. Keep your hands soft, avoiding any tension or squeezing.

3. Apply light pressure: Apply enough pressure to naturally stretch the skin as far as it will go, and to direct fluid upward to the lymph nodes (in the neck, armpits, groin, etc.). The pressure should be gentle; there should be no deep pressure or pain.

4. Lift your hand: Lift your hand to allow the skin to return to its natural state. This lift should feel smooth; there should be no discomfort or pulling. Please wait for the skin to return to its original state before repeating.

5. Work upwards: Work in small sections of the body, always directing the fluid upwards towards the lymph nodes. Repeat for 5-7 repetitions on each area, then move on to the next.

6. Listen to your body: if you feel any discomfort, reduce the pressure. If you see redness or feel the muscles under your hand, you may be pressing too hard.

7. Finish calmly: When you are finished, practice diaphragmatic breathing. This allows the body to process and integrate the lymph flow.

Important Notice: The following arm massage techniques are designed for lymphedema in the left arm. If you have lymphedema in your right arm, please apply the techniques in the reverse direction.

●: This red circle represents the arm with lymphedema.

Step 1: Diaphragmatic Breathing

Starting with diaphragmatic breathing helps to stimulate the lymph system in your whole body.

- Place the flat parts of your palms/hands on your stomach.
- Initially release all your breath through your mouth.
- Slowly, take a deep breath in through your nose (as if you were smelling flowers) and allow your abdomen to expand.
- Exhale slowly by pursing your lips (as if you were about to blow out a candle).
- Do not tighten your abdomen during exhalation.

Repeat 5-7 times.

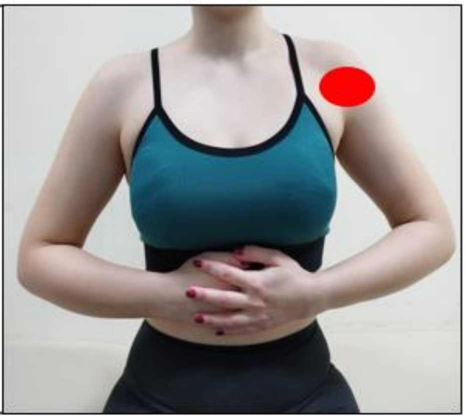

Step 2: Neck Lymph Drainage

- Place the inner surface of your fingers under your ear.
- Draw an inverted letter 'ㄱ' backwards towards your neck (away from your face) and downwards towards your heart.
- Following this rhythm, try to slowly, gently massage your neck with your hands still.

Repeat 5-7 times.

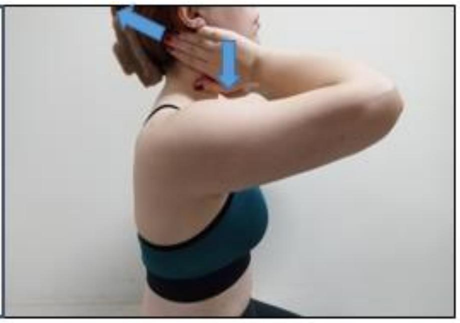

Step 3: Neck Lymph Drainage (Continues)

- You can massage 1 side or both sides simultaneously, cross your hands if you do both.
- Put the flat parts of your fingers on both sides of your neck, just above your collarbone.
- Make a shrugging motion to find the right point and feel the depression in the skin, your hand should be at this point.
- Massage downwards and inwards towards your collarbone.
- As far as your skin will naturally go, gently stretch and release.

Repeat 5-7 times.

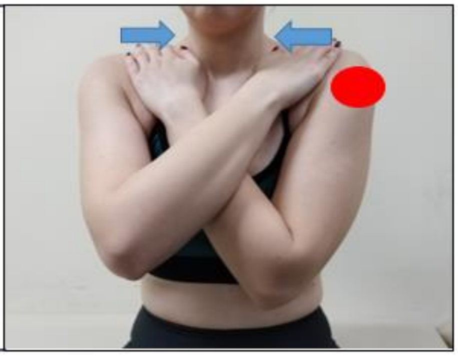

Step 4: Neck Lymph Drainage (Continues)

- Place your fingertips on either side of your neck in a slightly bent position.
- Stretch and relax your skin by slowly sliding your hands inwards and towards the hollow where your collarbones meet.
- The massage will look like two '**J**' strokes facing each other.

Repeat 5-7 times.

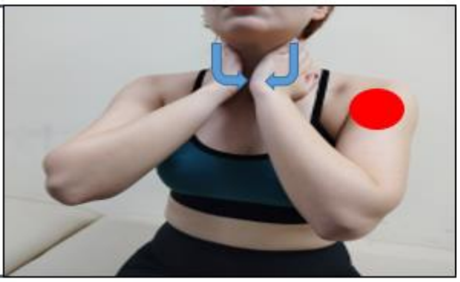

Step 5: Armpit Lymph Node Stimulation

- Place the inner surfaces of your fingers in the crease of your armpit on the side of your body with lymphedema.
- Gently pull upward and inward toward your body, then release.
- Repeat this motion slowly, ensuring that you don't apply too much pressure.

Repeat 5-7 times.

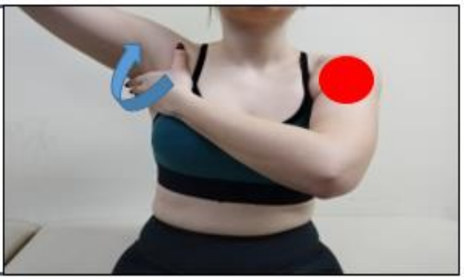

Step 6: Lymphatic Fluid Diverted from the Lymphedematous Side to the Healthy Side

- Line up the centre of your chest.
- First place your hand from this centre line to the side closer to your healthy arm (away from the arm with lymphedema).
- Without lifting your hand, stretch your skin upwards and sideways.

Repeat 5-7 times.

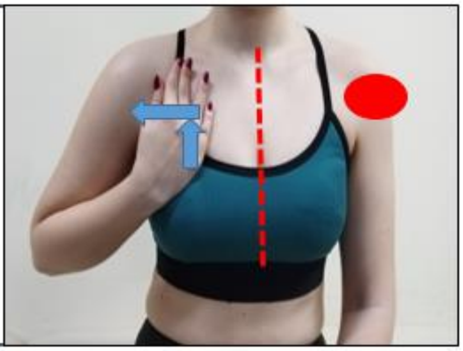

Step 7: Lymphatic Fluid Diverted from the Lymphedematous Side to the Healthy Side (Continues)

- Line up the centre of your chest.
- Place your hand on the side of this centre line close to the arm with lymphedema (away from the healthy arm).
- Without lifting your hand, stretch your skin in an upward and sideways direction.

Repeat 5-7 times.

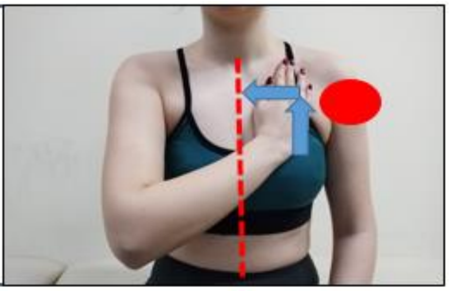

Step 8: Lymphatic Fluid Diverted from the Lymphedematous Side to the Healthy Side (Continues)

- Place your hand on the side close to the arm with lymphedema.
- With the inner surface of your hand, apply directive pressure upwards and towards the intact side, moving towards the armpit of the intact side.
- After stretching the skin upwards and towards the intact side with each stimulus, remove your hand and allow the skin to return to its original position.

Repeat 5-7 times.

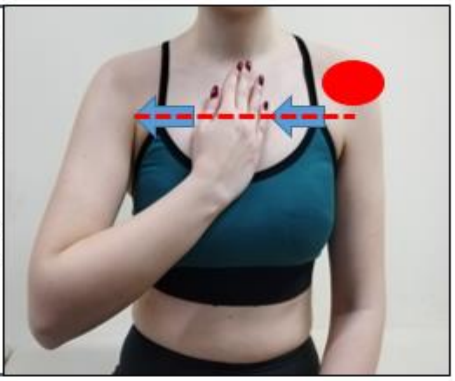

Step 9: Groin Area Lymph Node Stimulation

- To stimulate the lymph nodes in your groin area, place the flat surfaces of your fingers just below your waist, where your leg meets your body.
- This is the crease of your groin.
- Gently press your fingers into the skin and then slowly move them up and inwards, towards the centre of your body.
- Release the pressure and allow the skin to return to its original position.

Repeat 5-7 times.

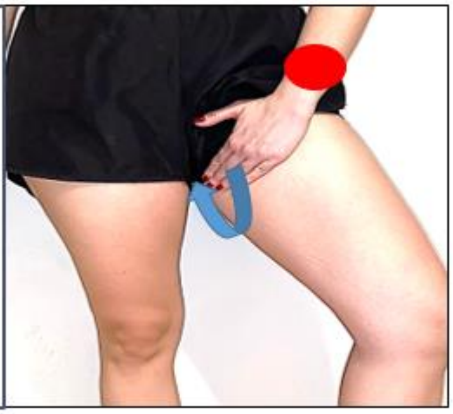

Step 10: Diversion of Lymphatic Fluid from the Lymphedematous Arm to the Healthy Groin Lymph Node Side

- Place your hand under your armpit on the side with lymphedema.
- Move from your armpit to the groin on the affected side.
- Work your way downwards and backwards, applying pressure.
- Be careful to move slightly obliquely from the armpit to the groin.
- Gently stretch the skin as far as it can naturally go.
- Then release the pressure and allow the skin to return to its original position.

Repeat 5-7 times.

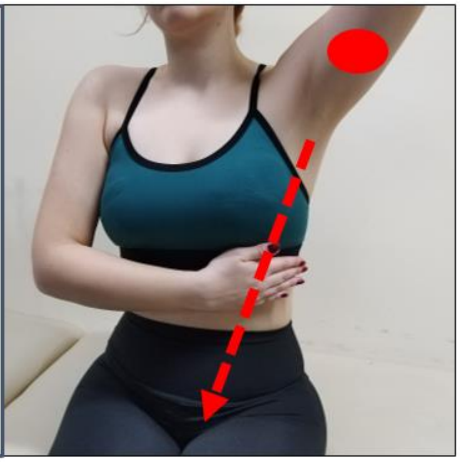

Step 11: Lymphedema Arm Massage

Warning: Before massaging your lymphedema arm, support your arm with a pillow.

The fluid in your arm with lymphedema normally drains through your arm to the lymph nodes in your armpit. After breast cancer treatments, the fluid collects in your arm because your lymph nodes are damaged. Your aim is to transfer this fluid to your healthy lymph nodes. You will start from the inside of your arm and transfer the fluid there to the back of your arm, then you need to transfer the fluid from the back of your arm to the neck and healthy side lymph nodes. In this way you will make a gradual transport.

Let's start our arm lymphedema massage step by step!

- Place your hand on the inside of your arm (near the elbow).
- Starting from the inside of your arm, stretch and release the skin towards the outside.
- Your hand stretches should be directed upwards and outwards respectively.
- Gradually work your way up to the upper part of your upper arm.

Repeat 5-7 times.

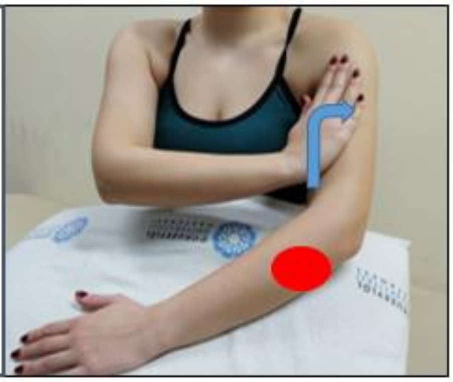

Step 12: Lymphedema Arm Massage (Continues)

- Place your hand behind the outside of your arm (near the elbow).
- Starting from the outside of your arm downwards, move upwards to the shoulder (including the shoulder).
- Your hand stretches should be outwards and upwards respectively.

Repeat 5-7 times.

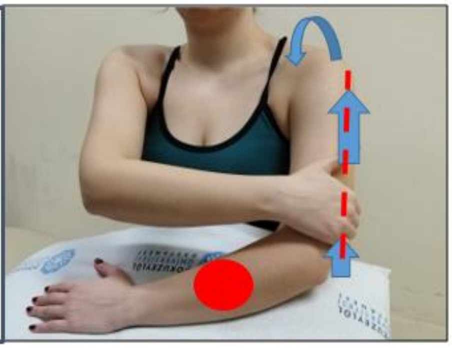

Step 13: Lymphedema Arm Massage (Continues)

- Turn your palm upwards.
- Place your hand on the inside of your elbow.
- Without lifting your hand, draw the letter '**L**' outwards and upwards in the elbow area.

Repeat 5-7 times.

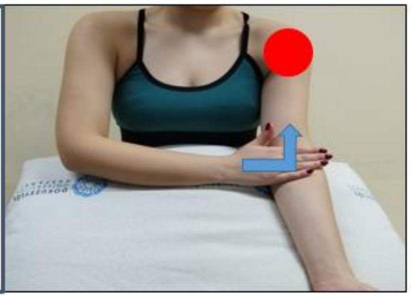

Step 14: Lymphedema Arm Massage (Continues)

- Place your hand just above the wrist.
- With the inside surface of your hand, apply guiding pressure to the outside of the arm and upwards, moving upwards up the arm.
- After stretching the skin back outwards and upwards with each stimulus, remove your hand and allow the skin to return to its original position.

Repeat 5-7 times.

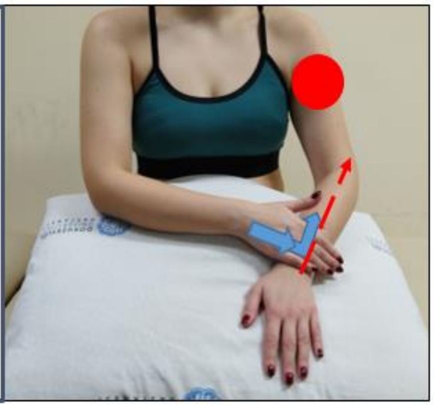

Step 15: Lymphedema Arm Massage (Continues)

- Turn your palm up.
- Place your hand just above your wrist.
- Apply guiding pressure with the inside surface of your hand outwards and upwards on the arm.
- Move upwards towards the part of the arm up to the elbow.
- After stretching each skin outwards and upwards, pull your hand away and allow the skin to return to its original position.

Repeat 5-7 times.

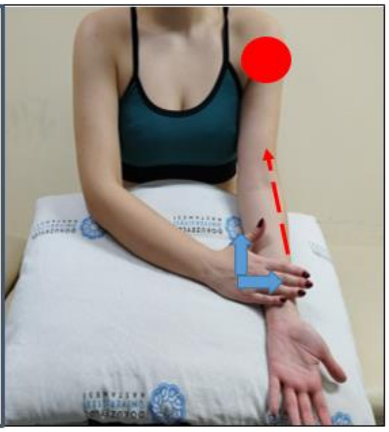

Step 16: Lymphedema Arm Massage (Continues)

- After directing the fluid up to the elbow, it is necessary to apply again from the outer side of the arm to the shoulder in order for the fluid to drain into the lymph channels.
- Place your hand behind the outside of your arm (close to the elbow).
- Starting from the outside of your arm, move downwards, upwards towards the shoulder (including the shoulder).
- Your hand stretches should be outwards and upwards respectively.

Repeat 5-7 times.

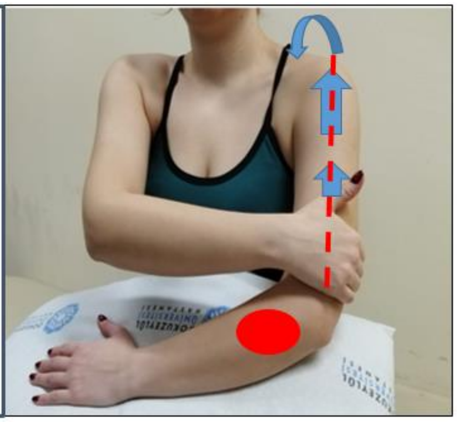

Step 17: Lymphedematous Hand Massage

- Without lifting your hand, apply outward and upward pressure on the top of the hand.
- After stretching the skin outwards and upwards with each stimulus, remove your hand and allow the skin to return to its original position.

Repeat 5-7 times.

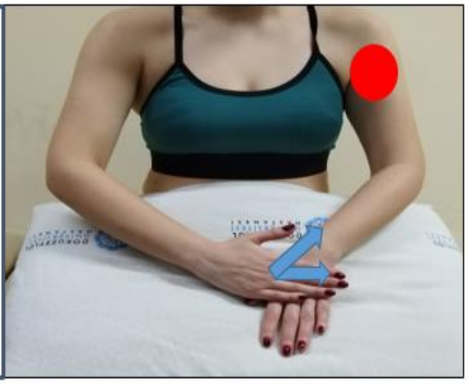

Step 18: Finger massage of the Lymphedematous Hand

- Place the index finger and thumb of your affected hand at the base of your swollen finger.
- Gently stretch and release the skin against your palm and massage your finger.
- Massage each finger one at a time.

Repeat 5-7 times.

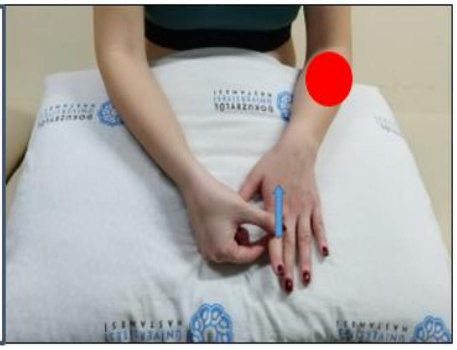

Step 19: Diaphragmatic Breathing

- Finishing with diaphragmatic breathing enables the body to process and integrate the lymph flow.
- Place the flat parts of your palms/hands on your stomach.
- Initially exhale all your breath through your mouth.
- Slowly take a deep breath in through your nose (as if you were smelling flowers) and allow your abdomen to expand.
- Exhale slowly by pursing your lips (as if you were about to extinguish a candle).
- Do not tighten your abdomen during exhalation.

Repeat 5-7 times.

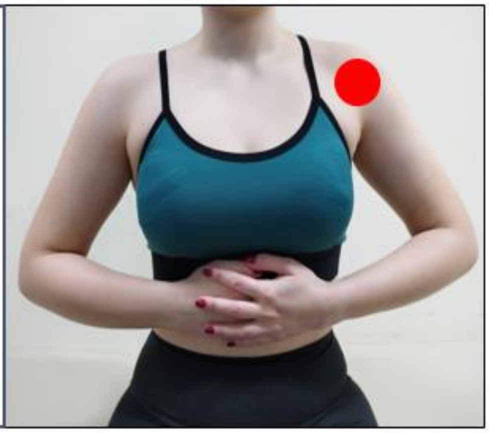

Chapter 3: Multilayer Short Traction Bandage Application

How Should I Do Self Bandage for My Arm Lymphedema?

This chapter explains:

1) What is self-bandage and why is it important?

2) How to perform self-bandage step by step?

Warning: This booklet is personalized for you, do not recommend it to patients who have not had a pre-consultation with a lymphedema therapist.

1. **What is self-bandage and why is it important?**

- Self-bandaging is a technique used to manage lymphedema by applying compression bandages to the affected limb. When applied after manual lymph drainage, it prevents the transported fluid from refilling the arm. In general, it is the key stage of treatment. It helps to reduce swelling and prevent fluid accumulation by increasing pressure on the tissue.

2. **How to perform self-bandage step by step?**

Warning: If you feel bruising or numbness/tingling in your fingertips a few hours after bandage application, remove the bandage.

Open the bandage if you feel pain in your arm.

Apply multi-layered cotton wool to prevent the bandage from irritating your skin.

1. **Prepare Your Arm:** Make sure your skin is clean and dry. If your skin feels dry, apply a light, oil-free moisturizer and wait for it to fully absorb before applying the bandage.

2. **Position Your Arm:** Sit comfortably in a relaxed position. It’s important to elevate your arm slightly, using a pillow or cushion.

3. **Support Sensitive Areas:** If necessary, place soft padding around sensitive areas such as your elbow or wrist. If you feel discomfort in the bony bump, apply the same application to these areas. This helps prevent discomfort and ensures that the pressure from the bandage is evenly distributed.

4. **Start at the Wrist (Distal End):** Begin applying the bandage at the furthest point of your arm—around your wrist. Hold the bandage firmly but gently, ensuring it lays flat against your skin as you apply it.

5. **Apply Consistent, Light Pressure:** Continue applying the bandage in a spiral pattern, moving upwards from the wrist to the forearm, and then toward the upper arm. Each layer of the bandage should overlap the previous one by about half the width of the bandage, ensuring even compression.

6. **Firm but Comfortable Pressure:** The bandage should feel firm enough to provide compression but should not cause any pain, numbness, or tingling. Check the pressure regularly to make sure it's comfortable.

7. **Gradually Decrease Pressure as You Move Up:** The compression should be strongest at the wrist or fingers and gradually decrease as you move towards the elbow and shoulder. This gradient compression helps the lymph fluid move upward toward the central lymphatic system.

8. **Check and Adjust the Bandage:** On each layer, find the point where the previous bandage depressurized before applying the next. Continue wrapping upwards starting from this point. This ensures consistent and even compression throughout the arm.

9. **Secure the bandage:** Secure the end using a plaster or other method instructed by your healthcare professional. Make sure the bandage stays securely in place.
10. **Check for Comfort:** After securing the bandage, check that it feels firm and consistent but not tight. You should feel compression without pain, and your arm should still move freely without numbness or tingling. If you feel any discomfort, loosen the bandage and reapply it with less tension.

Materials You Will Need:

- Gauze for fingers
- Cytokinet
- Cotton
- Compression bandages (short traction bandage)
- Plasters
- Padding (optional for extra comfort or protection)

Consult your lymphedema therapist before purchasing the materials. Ask about the quantity and suitability of the materials for you.

Step 1. Finger Gauze Wrapping on Lymphedematous Hand

- Wrap the gauze loosely around your wrist **(Figure 1a)**.
- Then bring the bandage under the nail of your thumb. Starting from the nail of your thumb **(Figure 1b)**, wrap it two or three times (depending on the size of your finger) towards the base (**Figure 1c**).
- Each layer of gauze should overlap about half the width of the previous one and provide an even compression.
- Do not pull the gauze too tightly when wrapping your thumb and fingers.
- Wrap the gauze around your wrist again.

Warning

- Do not cut the gauze.
- Do not wrap the gauze around the palm of your hand, but return to the wrist after each finger wrap.
- Do not make multiple layers at the same point on the wrist, avoid these multiple layers by wrapping the gauze from the wrist upwards.

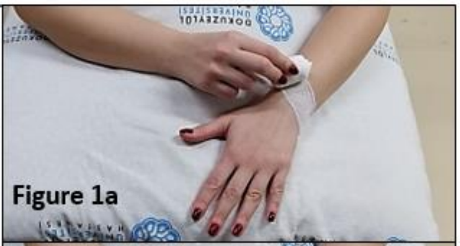
Figure 1a

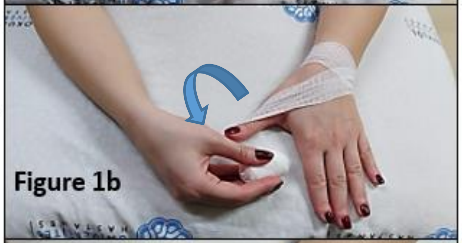
Figure 1b

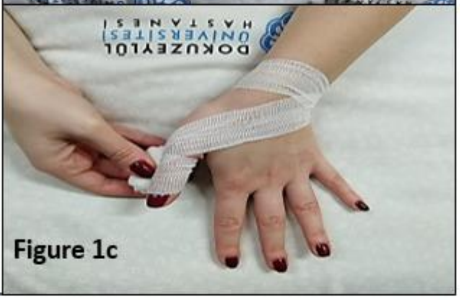
Figure 1c

Step 2. Finger Gauze Wrapping on Lymphedematous Hand (Continues)

- After finishing the thumb, move on to the index finger.
- Starting at the nail of the index finger, wrap two or three times (depending on the size of your finger) towards the base and continue wrapping each finger in the same way **(Figure 2a)**.
- Each layer of gauze should overlap about half the width of the previous one and provide an even compression.

Warning

- Do not cut the gauze.
- Do not wrap the gauze around the palm of your hand, return to the wrist after each finger wrap **(Figure 2b)**.
- Do not make multiple layers at the same point on the wrist, avoid these multiple layers by wrapping the gauze upwards from the wrist **(Figure 2c)**.
- Wrap all fingers using two pieces of gauze.

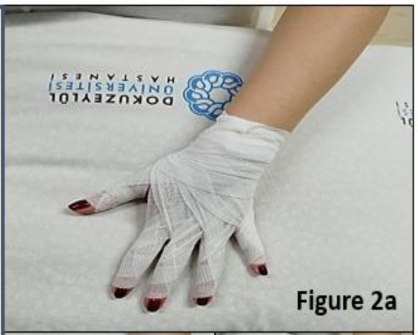
Figure 2a

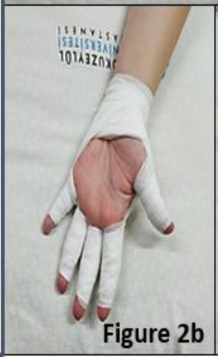
Figure 2b

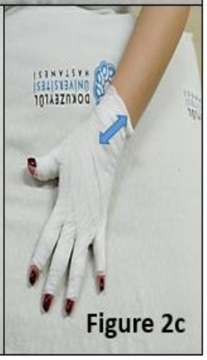
Figure 2c

Step 3. Wear the Cytokinet on Your Arm with Lymphedema

- Place the cytokinet over your arm.
- The length of the cytokinet should cover your hands and reach just below your armpit.
- Make a small hole in the cytokinet for your thumb and pass your thumb through it.

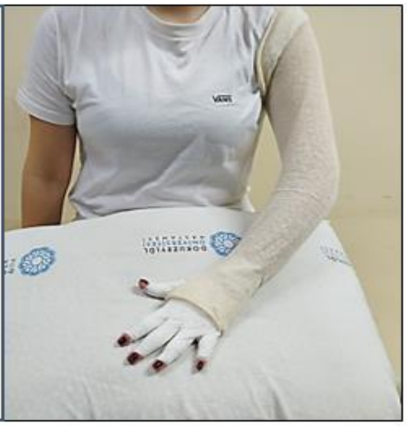

Step 4. Cotton Wrapping on the Arm with Lymphedema

- Wrapping cotton wool over the cytokinesis prevents irritation of your arm from the short traction bandage and ensures that the pressure is evenly distributed.
- Continue to wrap your arm with cotton wool until it forms a cylinder.
- At this stage, wrap the cotton without applying any tension.
- Starting at the base of your fingers, wrap it around your hand (your fingers should remain open) **(Figure 3a)**.
- Continue wrapping your palm towards your wrist.
- Cover your entire hand.
- Overlap the cotton in half with each wrap **(Figure 3b)**.
- Continue wrapping along the arm in the same way.
- Continue wrapping the cotton until you reach just below your armpit (about two fingers below your armpit).

Warning

Do not use tape as this can damage the cotton.

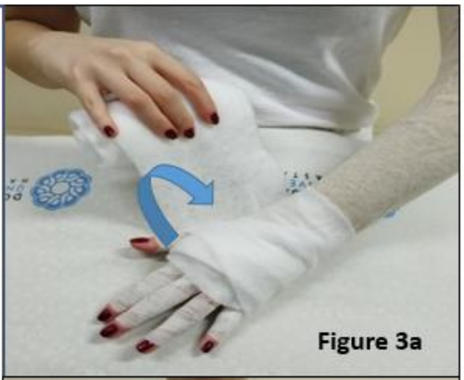

Figure 3a

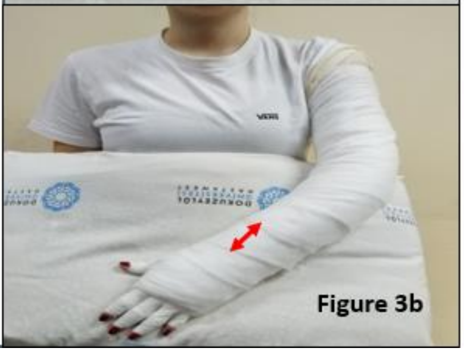

Figure 3b

Step 5. Short Traction Bandage Wrap Application to Arm with Lymphedema

- Start wrapping with a 6 cm bandage first.
- Do not pull the bandage wrap away from the bandaged area, keep the bandage wrap closed **(Figure 4a)**.
- Hold the bandage roll so that the bandage roll is on top **(Figure 4a)**.
- Spread your fingers wide when bandaging your hand (this will allow your fingers to move freely when bandaging your hand).
- Loosely rotate the 6 cm bandage around your wrist one turn (this will secure the bandage) **(Figure 4a)**.

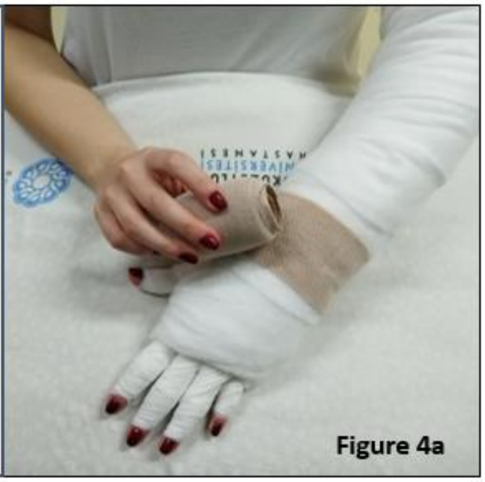

Figure 4a

Step 6. Short Traction Bandage Wrap Application to Arm with Lymphedema (Continues)

- After fixing the bandage one turn on your wrist, pull it from the top of your hand towards the base of your finger, wrap the bandage two to three more times, pulling it over the top of your hand and across the palm.
- Now wrap the bandage around your wrist and around your hand two or three times (in a figure 8 pattern).
- Apply an even pressure by pulling the bandage slightly every half turn.
- When the 6 cm bandage wrap is finished, fix the bandage with the help of a plaster.

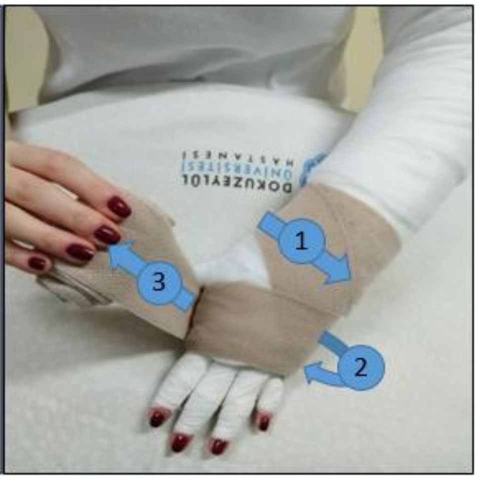

Step 7. Short Traction Bandage Wrap Application to Arm with Lymphedema (Continues)

- When you start bandaging the forearm (from the wrist to the elbow), make a fist with your hand. This activates the muscles in your forearm and prevents these bandages from putting extra pressure.
- Take an 8 cm bandage.
- Check the bandage you wrapped earlier by squeezing it with your other hand and start the 8 cm bandage where the bandage feels soft.
- Apply even pressure, pulling the bandage slightly every half turn **(Figure 5a)**.
- Overlap the bandage by half for each wrap (about two fingers) **(Figure 5b)**.
- When the bandage is finished, fix it with a plaster.

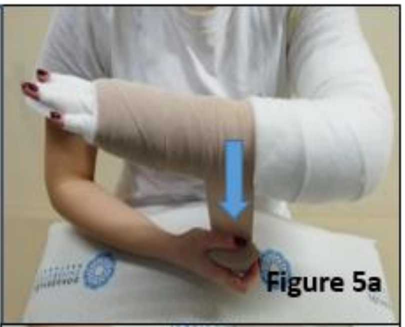

Figure 5a

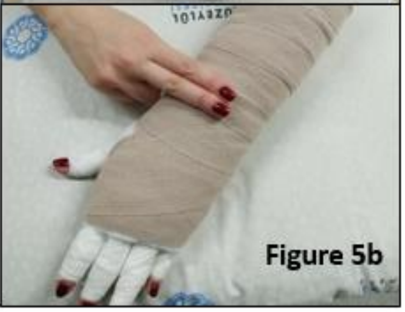

Figure 5b

Step 8. Short Traction Bandage Wrap Application to Arm with Lymphedema (Continues)

- The third bandage can be 8 cm or 10 cm.
- Start wrapping the bandage where the bandage feels soft. This bandage should reach to the top of your arm.
- Overlap the bandage by half with each wrap.
- Pull the bandage slightly every half turn to ensure even pressure.
- Continue wrapping from the top of your arm to just below your armpit and tape the end of the bandage to prevent it from loosening.

Repeat this process until the bandage pressure is evenly distributed upwards.

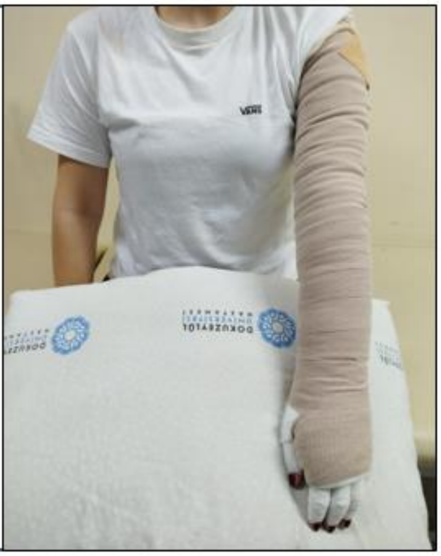

Warning: Always check your arm when you have finished wrapping your arm

Blood circulation check: Press on your fingernail. The red colour should disappear or fade.

The colour should return a few seconds after you remove the pressure.

If this does not happen, you may have bandaged it too tightly.

You will need to remove the bandage and wrap it more loosely.

Chapter 4: Exercises

Which Exercises Should I Do for My Arm Lymphedema?

This chapter explains:

1) Why is it important to exercise for lymphedema?

2) How do I do the exercises step by step?

> ***Warning:*** This booklet is personalized for you, do not recommend it to patients who have not had a pre-consultation with a lymphedema therapist.

1. Why is it important to exercise for lymphedema?

- Exercise is really important for managing lymphedema. The lymphatic system is a circulatory system and transports fluid through the contraction of muscles. Exercise is helpful for the lymphatic fluid circulating in your body to return back to the heart. When you move, your muscles pump and this helps to move the extra fluid that accumulates in your arm, which reduces swelling.

2. How do I do the exercises step by step?

> **Warning:** Make sure you do breathe control and listen to your body during the exercises.

> **Warning:** Stop exercising if you experience excessive sweating, fatigue or pain during exercise.

1. **Find a comfortable setting:** Do your exercises in a well-ventilated environment, away from distractions. Make sure your body is relaxed. Start with diaphragmatic breathing, which helps to increase lymph flow.

2. **Wear comfortable and breathable fabric:** Do not wear tight clothing that will impede circulation during exercise. Do your exercises in comfortable cotton clothes.

3. **Focus on Fluid Movement, Not Intensity:** Aim for controlled, smooth exercises to encourage lymph flow without straining your muscles.

4. **Breathe in to Start the Movement, Breathe out to Complete It:** Inhale deeply at the start of the exercise, then exhale slowly as you finish the movement, aiming for a longer exhalation than inhalation.

5. **Start from the Foot (Distal End):** Start the exercise from the ankle. The reason for this is to accelerate the lymphatic flow of the whole body and create a pump effect.

6. **Work Within Your Comfort Zone:** Stay mindful of your limits—if you feel pain or discomfort, stop and rest.

7. **Hydrate:** Drink plenty of water to support lymphatic function.

8. **Finish with Deep Breathing for Lymphatic Support:** End your session with diaphragmatic breathing to help your body process the fluid flow.

Tips:

Start slow: Always perform exercises slowly and with breath control.

Use compression: The use of bandages, always do the step of wrapping a bandage around your arm before exercising.

Exercise frequency: Perform all exercises ***once a day*** for ***two sets*** of ***five to seven repetitions.***

Step 1: Diaphragmatic Breathing

Starting with diaphragmatic breathing helps to stimulate the lymph system in your whole body.

- Place the flat parts of your palms/hands on your stomach.
- Initially release all your breath through your mouth.
- Slowly, take a deep breath in through your nose (as if you were smelling flowers) and allow your abdomen to expand.
- Exhale slowly by pursing your lips (as if you were about to blow out a candle).

Repeat 5-7 times.

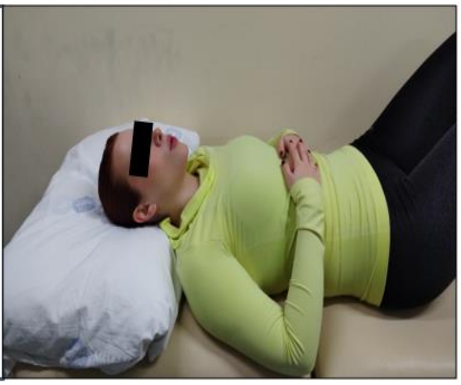

Step 2: Ankle Pumping Exercise

- Start by lying down with your legs in a comfortable position.
- Start by lifting the toes of one foot towards your shin and slightly stretching your ankle (flexing your calf). At the same time, lower the toes of the other foot towards the floor (stretch well).
- Then alternate the movement so that one ankle is pointing up while the other is pointing down. This creates an alternating ‘pumping’ movement with a smooth rhythm.

Repeat 5-7 times/ two sets.

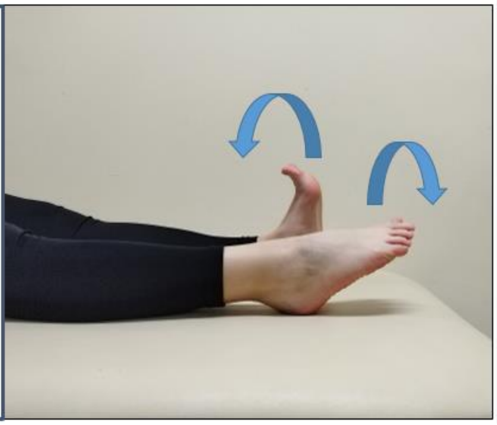

Step 3: Hip Flexor Stretching Exercise

- Start by lying on your back with your knees bent and your feet flat on the floor. Relax your arms at your sides.
- Pull one knee gently with your hands and slowly bring it towards your chest. Keep the other foot flat on the floor while stretching.
- Hold the position for about 15-20 seconds.
- Release your knee and put your foot back on the floor, then repeat with the other leg.

Repeat 5-7 times/two sets.

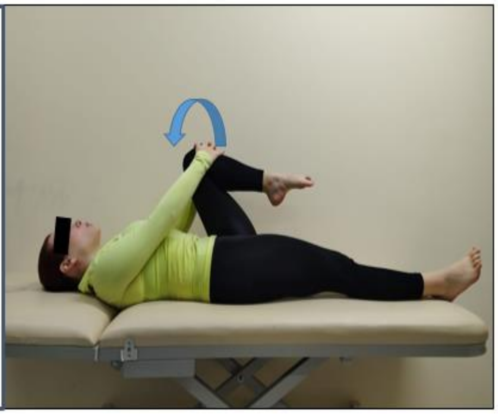

Step 4: Lumbar Stretching Exercise

- Start by lying on your back with your knees bent and your feet flat on the floor.
- Relax your arms at your sides.
- Gently pull both knees with your hands and slowly bring them towards your chest.
- Hold the position for about 15-20 seconds.
- Release your knees and place your feet back on the floor.

Repeat 5-7 times/two sets.

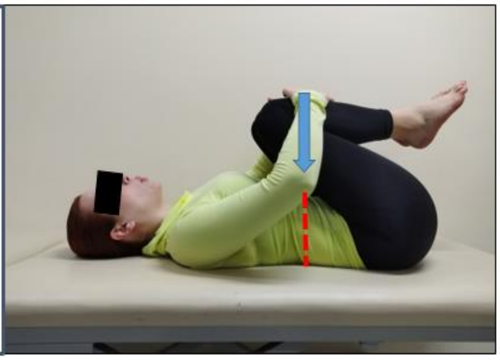

Step 5: Pelvic Bridge Exercise

- Your arms should be relaxed at your sides with your palms facing down.
- Slowly engage your core and squeeze your glutes, then slowly lift your hips off the floor.
- In the last part of the movement, aim to create a straight line from your shoulders to your knees (your spine should be straight) and your angle with the floor should be 90 degrees.
- Hold the position for a few seconds, making sure your glutes and core are engaged.
- Slowly lower your hips to the floor.

Repeat 5-7 times/two sets.

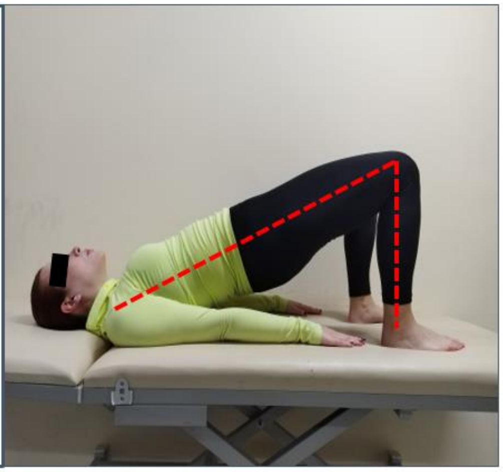

Step 6: Lumbar Rotation Stretching

- This movement is a good exercise to activate the lymphatic pathways of the trunk.
- Lying on your back, lie hip-width apart with your knees bent and your feet flat on the floor.
- Slowly lower your knees to one side while keeping your shoulders flat on the floor. Try to keep your knees together as your lower back and hips rotate.
- Simultaneously with your hip movement, rotate your arm and head in the opposite direction of your hip rotation.
- Hold the position for 15-20 seconds, feeling the tension in your lower back and torso.

Repeat 5-7 times/two sets.

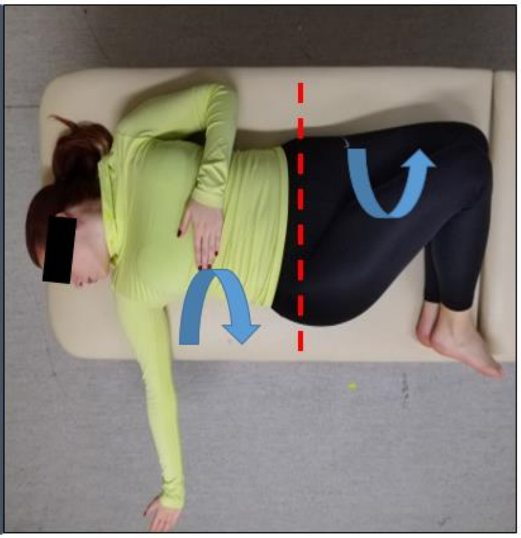

Step 7: Hand Compression Exercise

- This exercise helps stimulate fluid movement in your arms by using gentle pressure.
- Sit in a relaxed position, keeping your shoulders relaxed.
- Bring both hands together in front of your chest, palms facing each other.
- Slowly press the palms of your hands together, applying even pressure without straining. You should feel some resistance but not pain.
- Maintain the pressure for a few seconds, focusing on feeling the gentle stretch and activation in your arms.
- Slowly release the pressure and let your hands relax for a moment.

Repeat 5-7 times/two sets.

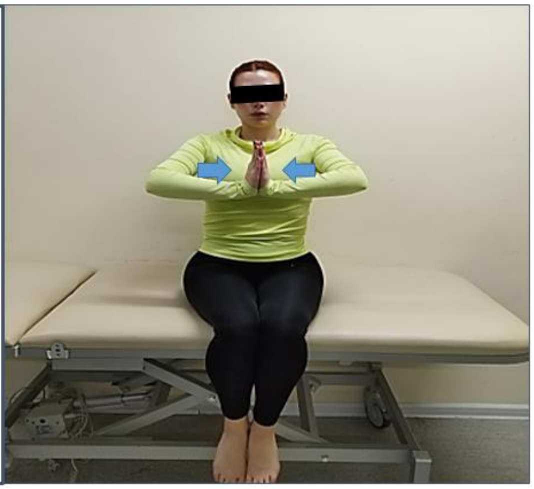

Step 8: Hand Pumps with Arms Elevated Forwards

- Sit with your back straight and shoulders relaxed.
- Raise both arms in front of you to shoulder height with your elbows straight and palms facing the floor.
- Make a fist, feel the pressure in your palms, and then release.
- Keep the movement smooth and controlled.
- Take your time, squeeze your hands, wait 5 seconds, open them, feel the tension in your palms, wait and make a fist again.

Repeat 5-7 times/two sets.

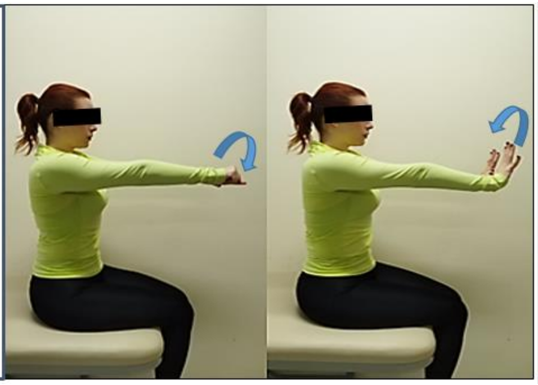

Step 9: Hand Pumps with Arms Elevated to Side

- Sit with your back straight and shoulders relaxed.
- Raise both arms by your side with your elbows straight and palms facing the floor (in the shape of the letter 'T').
- Make a fist, feel the pressure in your palms and then release.
- Keep the movement smooth and controlled.
- Take your time, clench your hands, wait 5 seconds, open, feel the tension in your palms, wait and make a fist again.

Repeat 5-7 times/two sets.

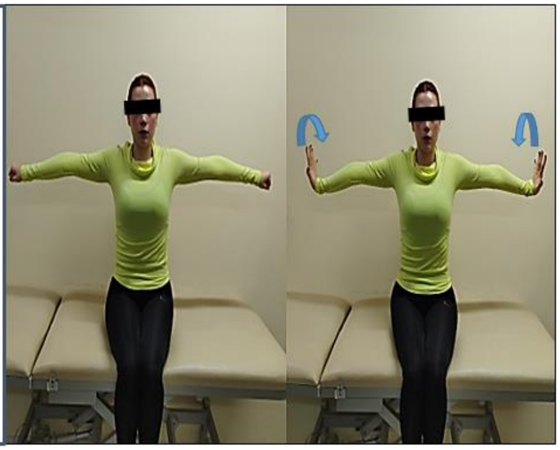

Step 10: Hand Pumps with Arm Elevated Upwards

- Sit with your back straight and shoulders relaxed.
- Raise both arms up with your elbows straight.
- Make a fist, feel the pressure in your palms (hold for 5 seconds).
- Open your palms and stretch your palms as if you were reaching up.
- Keep the movement smooth and controlled.

Repeat 5-7 times/two sets.

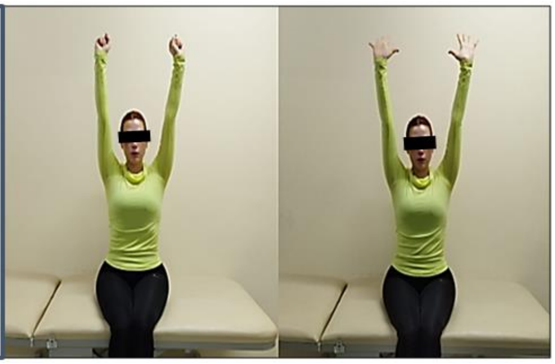

Step 11: Arm Hug Exercise

- Sit with your back straight and shoulders open.
- With your elbows straight, raise both arms out to the side until they are at shoulder level.
- With both arms, gently hug your arms around your body as if you were hugging yourself.

Repeat 5-7 times/two sets.

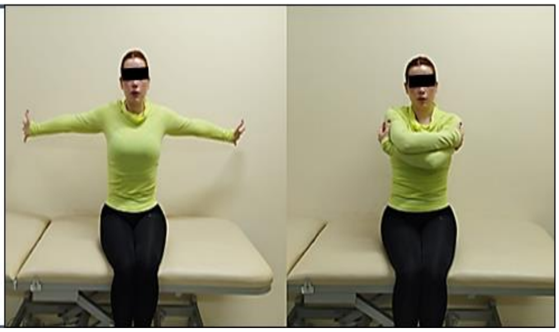

Step 12: Dynamic Arm Stretching Exercise

- Sit with your back straight, ensuring a relaxed but upright position.
- Keep your elbows straight and lift both arms in front of you until they reach shoulder height.
- Rotate your arms so your palms are facing the ceiling.
- Close your hands into fists, with your fingers curled in.
- Gently bend your wrists towards your body.
- Slowly bend your elbows and bring them towards your body.
- Gradually start to straighten your elbows, extending them back to the starting position.
- As your elbows straighten, spread your wrists down towards the floor.
- Finally, open your fists and stretch your hands out as if reaching for something far away.

Repeat 5-7 times/two sets.

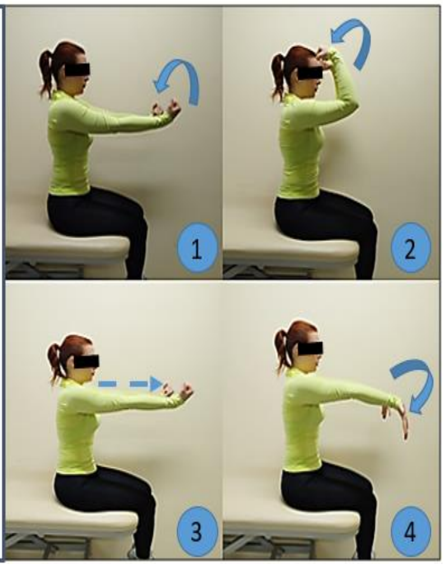

Follow-up Diary

Applications / Dates	Skin/nail Care	Manual Lymph Drainage	Bandage	Exercises	Notes
1st session					
2nd session					
3rd session					
4th session					
5th session					
6th session					
7th session					
8th session					
9th session					
10th session					
11th session					
12th session					
13th session					
14th session					
15th session					

Notes: This follow-up diary has been prepared to record which day you have done the applications. Remember to bring it with you to your control session and show it to your therapist.
